# Student's tutorial on bloom hypotheses in the context of phytoplankton annual cycles

**DOI:** 10.1111/gcb.13858

**Published:** 2017-09-22

**Authors:** Michael J. Behrenfeld, Emmanuel S. Boss

**Affiliations:** ^1^ Department of Botany and Plant Pathology Oregon State University Corvallis OR USA; ^2^ School of Marine Sciences University of Maine Orono ME USA

**Keywords:** annual cycles, biologic rates, biomass, phytoplankton blooms

## Abstract

Phytoplankton blooms are elements in repeating annual cycles of phytoplankton biomass and they have significant ecological and biogeochemical consequences. Temporal changes in phytoplankton biomass are governed by complex predator–prey interactions and physically driven variations in upper water column growth conditions (light, nutrient, and temperature). Understanding these dependencies is fundamental to assess future change in bloom frequency, duration, and magnitude and thus represents a quintessential challenge in global change biology. A variety of contrasting hypotheses have emerged in the literature to explain phytoplankton blooms, but over time the basic tenets of these hypotheses have become unclear. Here, we provide a “tutorial” on the development of these concepts and the fundamental elements distinguishing each hypothesis. The intent of this tutorial is to provide a useful background and set of tools for reading the bloom literature and to give some suggestions for future studies. Our tutorial is written for “students” at all stages of their career. We hope it is equally useful and interesting to those with only a cursory interest in blooms as those deeply immersed in the challenge of understanding the temporal dynamics of phytoplankton biomass and predicting its future change.

## 1 INTRODUCTION

Annual cycles in phytoplankton standing stocks differ dramatically across the global ocean. These cycles oscillate within a highly constrained range over the broad oligotrophic gyres, whereas they may be punctuated by massive phytoplankton blooms in coastal and high latitude regions. Despite their often ephemeral nature, blooms play a major role in ocean carbon biogeochemistry. In regions where blooms are predictably recurrent, these events have governed evolution in the lifecycles and migratory behaviors of organisms ranging from zooplankton to whales and birds (e.g., Cushing, 1969, 1990; Longhurst, 2007). In all cases, phytoplankton blooms are a consequence of ecosystem imbalances between phytoplankton division and loss rates, but they also show a clear dependence on abiotic factors influencing photosynthesis and division. These environmental factors include incident sunlight and its subsurface attenuation, surface layer mixing, nutrients, and temperature. Each of these physical and chemical properties is directly linked to climate. Consequently, a very real potential exists for global climate change to impact the timing, location, magnitude, and/or interannual variability of phytoplankton blooms, and thus ocean biogeochemistry and higher trophic level ecology and standing stocks.

Processes regulating phytoplankton blooms have been a topic of interest in the aquatic sciences for over a century. For my coauthor and I, a keen interest in blooms began with the work leading to the satellite‐based Behrenfeld (2010) and subsequent field‐based Boss and Behrenfeld (2010) publications. Our interest has only intensified in the years since, paralleled by a community‐wide resurgence of publications on blooms. In some respects, it seems that this flurry of activity has led to greater confusion, rather than clarity, regarding contrasting bloom hypotheses. This confusion is such that it is no longer clear what concept is being referred to when a given study purports to align with or even confirm a given hypothesis. One prerequisite that can be established for any valid bloom hypothesis is that its conceptual basis must not result in untenable conclusions regarding biomass dynamics at other times of the year. It is for this reason that bloom hypotheses are discussed here within the context of complete annual cycles.

The intent of this tutorial is to provide some clarity on bloom hypotheses and hopefully a helpful guide for reading the bloom literature. Time will tell if we have succeeded. Our tutorial begins with a brief overview of basic concepts that set the stage for the remaining discussion. We then turn the clock back to the early 1900s to revisit thoughts at that time regarding annual cycles in phytoplankton standing stocks. This recount provides important context for the later development of the Critical Depth Hypothesis and more modern explanations of annual biomass cycles within which blooms occur. It may come as a surprise to you to read below that the original Critical Depth Hypothesis was not intended to predict when a bloom will happen, but rather when it can or cannot happen. We then describe how the Critical Depth Hypothesis has metamorphosed over the years into a concept inconsistent with its original intent. This important change has gone unrecognized in the bloom literature during recent decades and, indeed, my coauthor and I have incorrectly described the Critical Depth Hypothesis in multiple publications (as have so many others). In an attempt to reduce confusion in future discussions and publications, we propose two new titles for the divergent meanings of the Critical Depth Hypothesis. The final sections of this tutorial overview the recent Disturbance‐Recovery Hypothesis and discuss potentially fruitful directions for future research. A gratifying aspect of preparing this tutorial was re‐reading some of the older literature, many quotes from which you will find in the text below. We hope this document proves to be useful, or at the very least an enjoyable read. So without further ado, let us set the stage with some basic concepts.

## 2 DEFINING THE PROBLEM

What causes phytoplankton biomass to change over time? The answer to this question is obviously a difference between the specific rates of phytoplankton division (μ; day^−1^) and loss (*l*; day^−1^) (Table 1). This imbalance is quantified by the specific rate of change in biomass (*r*; day^−1^):

**Table 1:** Summary of key terms and concepts discussed in this tutorial, along with notations, units, and explanatory notes

Term	Symbol	Unit	Notes
Phytoplankton biomass	*C*_phyto_	mg C m^−3^	Temporal changes reflect differences in division and loss rates when mixing depths are constant or shoaling
Integrated phytoplankton biomass	∑*C*_phyto_	mg C m^−2^	Temporal changes reflect differences in division and loss rates when mixing depths are deepening
Gross photosynthesis	GP	mg C m^−3^ day^−1^	Total diurnal carbon production
	GP*	day^−1^	*GP* normalized to *C*_phyto_
Phytoplankton respiration	*R*_phyto_	mg C m^−3^ day^−1^	Total diel carbon respiration
	*R* ^*^_phyto_	day^−1^	*R*_phyto_ normalized to *C*_phyto_
Net photosynthesis	NP	mg C m^−3^ day^−1^	Photosynthetic carbon production available for cell division (GP *−* *R*_phyto_)
	NP*	day^−1^	NP normalized to *C*_phyto_
Specific division rate	μ	day^−1^	See equation 2
Specific loss rate	*l*	day^−1^	Includes all processes that removed phytoplankton biomass from the mixed layer
Specific accumulation rate	*r*	day^−1^	Difference between μ and *l*; Quantifiable from changes in *C*_phyto_ when mixed layer depth is constant or shoaling and from changes in *EC*_phyto_ when mixed layer depth is increasing; Positive and negative values indicate increasing and decreasing phytoplankton populations, respectively

Term	Notes
Mixed layer depth	Depth of the surface layer where changes in temperature or density are less than a pre‐specified criteria (e.g., depth where density is 0.030 kg/m greater than the value at 10 m)
Mixing depth	Depth of active turbulent mixing, which may be equal to or less than the density‐defined mixed layer depth
Compensation depth	A conceptual depth horizon equivalent to the euphotic depth where, in the absence of vertical mixing, the time integrated light level over the diurnal period is sufficient for gross photosynthesis to exactly equal phytoplankton diel respiration. In a stratified water column, phytoplankton autotrophic cell division is only possible above the compensation depth. In a deeply mixing water column, the critical depth (see below) defines whether autotrophic cell division is possible
Critical depth	For a given incident light level (*PAR*) and diffuse attenuation coefficient (*K*_d_), the “critical depth” is the depth to which a phytoplankton population can be mixed such that, on average, the light absorbed for photosynthesis over the diurnal period is sufficient for gross photosynthesis to exactly equal phytoplankton diel respiration
Bloom	The qualitative condition of high *C*_phyto_. There is no objective and quantitative definition for a bloom (see text)
Blooming	The condition where μ* *> *l* and thus *r* has a positive value
Bloom initiation	The point in time where μ first exceeds *l* in a time series characterized by predominantly positive values of *r* and that eventually results in phytoplankton concentrations qualitatively considered to be a bloom. It should be noted that, in nature, short time scale variations in mixed layer growth conditions cause *r* to fluctuate between positive and negative values as *C*_phyto_ builds up to bloom concentrations
Benchmark annual cycle concept	The general concept of phytoplankton annual cycles and blooms developed from the late 1800s to early 1900s and synthesized by Gran (1932) and Atkins (1932) where winter mixing charges the surface layer with nutrients, water column stratification and increasing sunlight stimulate phytoplankton division rates and cause a bloom to begin, then further stratification leads to the bloom's demise until autumn mixing reintroduced nutrients and yields a secondary bloom until light levels once again become limiting
Critical depth hypothesis	A modification to the above benchmark concept where a specific threshold mixed layer light level has to be exceeded before *C*_phyto_ either can or will begin increasing (see below)
Gran and Braarud's critical photosynthesis hypothesis	The original *Critical Depth Hypothesis* introduced by Gran and Braarud (1935) where the threshold light level defined the condition where, on average for the mixed layer, GP* = R*_phyto_. This concept predicts when a bloom can happen, not when it does happen because it does not explicitly account for other loss processes
Critical division rate hypothesis	Modification of *Critical Depth Hypothesis* where the threshold light level defines the condition where, on average for the mixed layer, μ = *l*. This concept predicts when a bloom will happen and assumes that all loss processes can be predicted from mixed layer light conditions
Critical turbulence hypothesis	Modification of *Critical Division Rate Hypothesis* where growth conditions for phytoplankton are described for active mixing layer rather than a temperature or density‐defined mixed layer depth
Disturbance recovery hypothesis	Description of annual phytoplankton biomass cycles where phytoplankton division and loss rates are perpetually coupled, the sign and rate of change *C*_phyto_ is dependent on accelerations and decelerations in μ, blooms end when μ reaches its maximum value, and physical disturbances can cause μ* *> *l* even during periods of the year when μ is decreasing

(1) r=μ−l.

We can further expand the expression for μ as:

(2) $$\mu =\frac{1}{dt}\text{ln}\left(\frac{\text{NP d}t}{C_{\mathrm{phyto}}}+1\right)=\frac{1}{dt}\text{ln}\left(\frac{(\text{GP}-R_{\mathrm{phyto}})dt}{C_{\mathrm{phyto}}}+1\right)$$

where NP, GP, and *R*_phyto_ are phytoplankton net photosynthesis, gross photosynthesis, and respiration, respectively, (all with units of mg C m^−3^ day^−1^), dt is 1 day, and *C*_phyto_ is phytoplankton carbon concentration (mg C m^−3^). Adding an asterisk to denote normalization to *C*_phyto_, Equations (1) and (1) can be combined to:

(3) $$r=\frac{1}{dt}\text{ln}\left(\left({\text{GP}}^{*}-R_{\mathrm{phyto}}^{*}\right)dt+1\right)-l$$

Equation (2) summarizes the basic components of the bloom problem, which for this tutorial is restricted to blooms in the surface mixed layer. For phytoplankton biomass to increase in the mixed layer, the first (again, obvious) prerequisite is that the rate of phytoplankton daily gross photosynthesis must exceed the diel respiration of the phytoplankton. If this requirement is met (and assuming the mixed layer is not deepening, see below), then μ has a positive value but biomass might still decline if *l *> μ, where losses include grazing, mortality to viruses and bacteria, and sinking out of the mixed layer. Blooms result when μ exceeds *l* for a sufficient period of time for a significant accumulation in phytoplankton biomass to occur.

What is a “bloom”? Qualitatively, a bloom is a high concentration of phytoplankton (mg C m^−3^). Unfortunately, a quantitative definition for a bloom is a far more elusive problem. One approach for defining a bloom might be to establish a specific threshold criterion. For example, we could define a bloom as the condition where phytoplankton concentration exceeds, say, 40 mg C m^−3^, but this assignment would be a rather arbitrary “line in the sand.” For instance, it is easy to imagine two phytoplankton communities developing along identical temporal trajectories but one achieves a climax of 35 mg C m^−3^ and the other 40 mg C m^−3^. It is not defensible to conclude that the latter represents a bloom and the former does not. Defining a bloom by the rate of increase in phytoplankton is similarly problematic. For example, it is difficult to justify why a climax of 40 mg C m^−3^ achieved in 1 week is a bloom, but the same climax concentration achieved over 2 weeks or even 3 months is not a bloom. In general, definitions of blooms based on threshold criteria are inherently problematic and often lead to incorrect conclusions on when and where blooms occur and, more importantly, why they occur.

Interestingly, discussing and evaluating contrasting bloom hypotheses does not actually require a definitive quantification of what a bloom is. This is because the fundamental issue that bloom hypotheses attempt to explain is what conditions are necessary for phytoplankton biomass to accumulate. In other words, under what conditions is the sign of *r* positive? Answering this question obviously requires evaluating variations in *r* over time, yet a surprisingly large number of publications on blooms do not report rates of change in biomass. This failure compromises the validity of a given study's conclusions and is a significant contributor to the current confusion and debate in the bloom literature. We will discuss this issue in greater detail later, but for now the important point is that in this tutorial our primary concerns are variations in *r* over the annual cycle, factors that cause the sign of *r* to change from negative to positive (an event we refer to as “bloom initiation”), and the conditions under which the value of *r* is positive. This latter condition we refer to as “blooming,” irrespective of whether it ultimately leads to high phytoplankton biomass. For example, an early‐spring phytoplankton population increasing in biomass from 1 to 8 mg C m^−3^ (equivalent to a change in chlorophyll from 0.02 to 0.16 assuming a Chl:C ratio of 50) in a week is viewed here as an equivalent “blooming” event as a late‐spring population increasing from 10 to 80 mg C m^−3^ in a week. While only the latter case might be viewed as ending in concentrations associated with a “bloom,” both populations have the same specific rate of change in biomass (*r *= 0.3 day^−1^) and thus experienced equally favorable conditions for blooming. Focusing on the sign and magnitude of *r* makes the evaluation of blooming conditions objective and independent of initial biomass.

The final issue to address in setting the stage for this tutorial is what is meant by “biomass”? Our qualitative definition of a bloom implies that biomass is a volumetric property (i.e., mg C m^−3^), but it also states that a bloom is the consequence of μ exceeding *l*. This raises the complication that, under some conditions, μ can exceed *l* without an increase in phytoplankton concentration. A real‐world example of this is when convective mixed layer deepening dilutes a phytoplankton population at a rate equal to or greater than the excess of μ over *l*, causing the total mixed layer phytoplankton carbon (∑*C*_phyto_; mg C m^−2^) to increase but concentration to be either constant or decreasing. This scenario will clearly not yield what we qualitatively defined above as a bloom, but can it be considered “blooming” (i.e., positive *r*)? The answer to this question is not simply a matter of semantics, but is fundamental to understanding phytoplankton annual cycles. The following provides an example.

In the North Atlantic satellite‐based study of Behrenfeld (2010), the initial rise in phytoplankton concentration (mg C m^−3^) was found to coincide with the end of convective mixed layer deepening. Why? If we fail to consider how ∑*C*_phyto_ was changing prior to this transition, then we are left with the challenge of explaining why the end of convective mixing resulted in an abrupt change in the balance between μ and *l*. One proposed explanation is that the depth of active mixing (turbulence) is greatly reduced when convection stops and this change abruptly increases sunlight exposure in the actively mixing phytoplankton population such that μ exceeds *l* for the first time and *r* becomes positive. However, while the mixed layer physics of this explanation are sound and the proposed increase in μ with reduced turbulence is inevitable, an evaluation of ∑*C*_phyto_ during the convective mixing period reveals that μ exceeds *l* well before the end of convective mixing. In fact, Behrenfeld (2010) showed that ∑*C*_phyto_ can begin increasing in the subarctic Atlantic while incident sunlight is decreasing, mixing depth is increasing, and μ is still declining to its annual minimum. Thus, irrespective of whether one considers this winter increase in ∑*C*_phyto_ as “blooming,” recognizing the implications of these changes in terms of the balance between μ and *l* is essential for establishing the key drivers of annual phytoplankton biomass cycles and blooms. For this tutorial, we will use the term “biomass” when we mean mg C m^−3^ and the notation ∑*C*_phyto_ where it is important to consider depth‐integrated carbon.

Okay, so far, so good. The problem at hand is to understand what conditions allow phytoplankton biomass (mg C m^−3^) in the mixed layer to increase. We have noted that such increases must be sustained over a sufficient period of time to satisfy our qualitative definition of a bloom, but that the key issue of interest is the sign of *r*, which we will hereafter refer to as the “accumulation rate.” We have also noted that, in reading the bloom literature, it is important to pay attention to whether a given study specifically reports variations in *r* and, if mixing depths are increasing, addresses changes in ∑*C*_phyto_. We are now ready to step back in time to sample some of the developments in thinking regarding factors controlling phytoplankton biomass.

## 3 A DEVELOPING THOUGHT

An excellent and thorough account of early developments in thought on phytoplankton annual cycles has been provided by Mills (2012). This section of our tutorial is not intended to provide an equally exhaustive review, but only to highlight a select set of key developments relevant for critically evaluating the modern bloom literature. We have relied here on Mills (2012) for translating developments in non‐English publications.

Haaken H. Gran, who throughout his career made major contributions to our understanding of phytoplankton annual cycles, was the first to explicitly describe features of the Atlantic spring bloom and suggested that “such a universal phenomenon…must have a universal acting cause” (Gran, 1902). Excerpts from his “Pelagic Plant Life” chapter in the 1912 publication, “The Depths of the Ocean” (Murray & Hjort, 1912) provide a further benchmark on early thoughts regarding blooms. At this point, Gran already recognized that the dominance in phytoplankton communities changes continuously over the year due to seasonal environmental changes (light, temperature, etc.) and the physical advection of populations:

Our investigations at different seasons, both in coastal waters and in the North Atlantic, have shown us that the flora of each locality is constantly changing. One species succeeds another as month follows month, and different societies predominate in the same locality at different seasons.

Gran then notes that this development is “…much more irregular than it would be if merely such simple factors as warmth and light controlled production..” and that a fundamental inconsistency can exist with even the known direct effect of temperature on metabolism, such that “…as far north as the coast of Norway, we find it is not the hottest months of summer that the plankton attains its maximum, but in the early part of the spring or the end of autumn.” From these observations, he highlights seasonal nutrient availability as also playing an important role in biomass variability, consistent with ideas repeatedly espoused by Karl Brandt. In the open sea away from terrestrial nutrient sources, Gran further describes how temporal changes in nutrients are reliant on vertical transport mechanisms.

But out in the open sea, there is another important source of nutriment to be taken into account. Nathansohn has pointed out the pelagic animals are constantly taking nutritive matter down into the deep water, and that for the time being it is accordingly withdrawn from the plants, even though the metabolism of the animals and the action of bacteria liberate it once more in inorganic form. These nutritive substances may… accompany the ascending water‐masses where off‐shore winds bring about upwelling…and where the surface‐layers, becoming chilled, sink and make room for warmer layers from below.

He then goes on to describe how, consistent with processes established in freshwater systems (Wipple, 1899), the seasonal cycle of wind‐driven and convective mixing in the higher latitude open ocean will impart a strong seasonal cycle on surface layer nutrients that in‐part is consistent with the seasonal cycle in plankton biomass. At this point, and consistent with Nathansohn (1908), Gran viewed vertical mixing has having a positive role on phytoplankton growth through its influence on nutrients.

The final relevant point we extract from Gran's chapter regards the relationship between rates of phytoplankton division, loss, and accumulation. Mid‐winter minima in phytoplankton biomass were seen as consistent with minima in light and temperature, but an observed second minimum in mid‐summer posed a problem. Gran's initial hypothesis regarding this annual feature was that it resulted from nutrient limitation of phytoplankton division in summer. He then devised a clever experiment to directly determine phytoplankton division rates from the fraction of dividing *Ceratium* cells in early morning. The outcome of the experiment was that division rates were rather maximum in summer than diminished, yielding the following conclusions:

The number of individuals at any given moment depends not merely upon the rate at which production has taken place, but also upon how many have perished or been carried away; and the causes bring about diminution, which we perhaps term factors of loss, may vary without being in any way directly connected with the conditions of existence of the plankton.

The fact that we find … that the plankton is less abundant in the summer months than in the spring, does not necessarily imply any unfavorable change in the conditions of existence due to summer. … We were obliged, therefore, to abandon our original intention, which was to ascertain the importance of such conditions of existence as dissolved nutritive substances, and particularly nitrogenous compounds.

and, finally,

Plants which are reproduced by division must necessarily decrease rapidly whenever vigorous augmentation ceases, if animals are constantly consuming numbers of them.

Recognizing the term “conditions of existence” as meaning light, temperature, and nutrient conditions and the term “vigorous augmentation” as meaning rapid division rate, what these statements are saying is that loss rates (*l*) play a crucial role in the annual biomass cycle and can even cause the rate of change in biomass (*r*) to be independent of division rate (μ).

The parallel emergence of the subarctic Atlantic spring bloom and the annual greening of Europe was seen early‐on as indicative that seasonal changes in light and temperature were equally important to both events (Schütt, 1892). Lohmann (1908) suggested that bloom onset could be predicted from the product of light and temperature. However, for aquatic systems, it was also recognized that subsurface light attenuation is an important factor influencing water column photosynthesis. Wipple (1899) conducted the first investigation of this problem, suspending bottles of diatoms at various depths in lakes to determine how changes in light impacted metabolism. Gran (1915) focused further on evaluating the depth at which phytoplankton photosynthesis was equal to phytoplankton respiration, which we now refer to as the “compensation depth” or “euphotic depth.” Atkins (1925) drew additional attention to the role of incident light, noting that interannual changes in the timing of the spring bloom could be correlated to variations in early spring sunshine. Marshall and Orr (1927) refined this idea such that it was the total incident light, not only sunshine, that was important for bloom development.

A critical next step in understanding phytoplankton annual cycles was to expand the role of mixing beyond simply the supply of nutrients. In 1928, Atkins (1928) wrote:

Three other factors [beyond incident light], however, may affect the illumination to which diatoms are exposed … turbidity of the water, … the amount of light reflected at the surface, … [and] suitable conditions as regards illumination, nutrient salts, and temperature … In autumn the surface cooling sets up convection currents and produces a very thorough mixing of the water. While this is in progress diatoms multiplying near the surface will be carried down into regions of lesser illumination, so it is unlikely that an outburst will occur.

This idea was developed further by Gran from an evaluation Southern Ocean data. During an investigation in the Weddell Sea, it was noted that phytoplankton biomass did not significantly increase under conditions of deep mixing despite high nutrient concentrations and that only after surface freshening did a dramatic bloom occur (Gran, 1931):

The diatoms also move with the vertical movements of the water, and … therefore no accumulation is found in the illuminated zone, with the effect that the whole production is retarded, because too many of the diatoms sink below the balance depth of the photosynthesis … [When] vertical circulation is stopped and the sinking of the diatoms retarded … it is clearly seen, how the phosphates (and nitrates) are consumed in the surface layers as far down as the photosynthesis is effective

By 1932, the work of Gran and Atkins had established the basic elements for a description of the annual phytoplankton cycle in bloom‐forming temperate regions. Deep winter mixing was seen as critical for charging the surface layer with nutrients, but also prevented an accumulation of biomass because light was insufficient to overcome phytoplankton respiration. A degree of water column stratification was therefore necessary for net photosynthesis to occur and thus a bloom to begin. Stratification was also seen as one cause of the bloom's demise, as it separated the phytoplankton from their source of nutrients, potentially resulting post‐bloom in a nutrient‐limited low‐biomass condition. This situation could then be altered in autumn when mixed layer deepening reintroduced nutrients from depth and yielded a secondary bloom until light levels once again prevented growth (Atkins, 1932; Gran, 1932).

A final critical component was added by H. W. Harvey, who re‐introduced the importance of grazing losses (e.g., Gran, 1912; Lohmann, 1908). Harvey noted that the collapse of a phytoplankton bloom could occur in the presence of excess nutrients, during high phytoplankton growth rates, and corresponded to an increase in grazers. He also emphasized that at any point in time the net primary production of phytoplankton exceeded (often to great extent) the rate of accumulation in biomass, implicating a perpetual and important role of grazers. Thus, a more complete view of the phytoplankton annual cycle emerged, where (Harvey, 1937):

several factors which influence, and from time to time control, the production of phytoplankton can now be enumerated with some degree of certainty—the concentration of phosphate, and of nitrate, the illumination and temperature, the rate at which the organisms are being eaten by zooplankton, and the extent to which vertical currents carry them down beyond the level of sufficient light

The forgoing brief historic foray has identified some of the key early developments in thought on phytoplankton blooms and annual cycles. The next three sections focus on the origin of the Critical Depth Hypothesis by Gran and Braarud (1935), its evaluation by Gordon Riley, and finally its treatment by Sverdrup (1953).

## 4 GRAN AND BRAARUD'S BAY OF FUNDY PROJECT

In 1931, Haaken Gran was appointed to study the potential effects that a planned Cooper dam project might have on the productivity of the Bay of Fundy, particularly with respect to the phytoplankton. Trygrve Braarud was subsequently appointed as an assistant to the investigation in 1932. For the Bay of Fundy Project, the intent was to conduct monthly sampling cruises from winter through the following autumn and to include a sampling of the western Gulf of Maine as a contrasting environment. From its onset, the project met significant setbacks, beginning with:

Unfortunately a fire in March, 1932, at the Atlantic Biological Station, the base of the field work, destroyed the greater part of the equipment for this work. The result was that the collection of phytoplankton material was delayed…until the month of April,

and followed by:

Though it was desirable to continue the investigation through part of the next year, at least, cuts in the appropriations made it necessary to discontinue the field work after the first of October 1932.

Apparently, the challenges of conducting large field programs today are little different from those a century ago!

Despite the above setbacks, the Bay of Fundy Project yielded a detailed description of seasonal physical, chemical, and biologic properties of the region and uncovered a curious discrepancy (Gran & Braarud, 1935):

In the gulf of Maine a rich diatom (Thalassiosira) plankton grows in April and May, representing the richest production of the whole year… In the bay of Fundy the turbulence is much more marked, particularly along the coasts. Here, also, a rich diatom grows in April and May over the whole bay except along the New Brunswick coast, where the turbulence seems to prevent a rich growth even in spring… While during summer the production of phytoplankton in the surface layers in the gulf of Maine is mainly limited by the low contents of phosphate and nitrate, caused by marked stratification, we find quite the inverse situation in the bay of Fundy. Here, the phosphates and nitrates are, particularly in June, present in excess, but the phytoplankton is nevertheless poorer than in the gulf of Maine, and in June extremely poor.

To this “Bay of Fundy paradox,” Gran and Braarud also noted that:

The influence of [zooplankton grazing] is, unfortunately, very difficult to determine quantitatively. It must be considerable, but we have not data to calculate its effect. We can only say that according to the zooplankton observations, the zooplankton of the bay of Fundy is on the whole less abundant than in the gulf of Maine, and therefore the consumption is certainly not sufficient in itself to explain the extreme poverty of the phytoplankton of the bay of Fundy as compared with the gulf, particularly in June

In the context of Equation (2), the challenge thus facing Gran and Braarud was that differences in loss rates (*l*) between the Bay of Fundy and Gulf of Maine could not explain the poverty of phytoplankton in the former region, there was no reason to assume that phytoplankton respiration rates ($R_{\mathrm{phyto}}^{*}$ ) were different between regions, and both regions had similar incident light levels.

Clearly, the key to the “Bay of Fundy paradox” was the enhanced turbulence and how this was linked to Gran's long held interest in compensation depths (Gran, 1915). Recognizing first how increased mixing can lead to autumn declines in phytoplankton, Gran and Braarud note:

Thus, the question, whether violent turbulence may make the phytoplankton increase or decrease, must to a large extent depend on the thickness of the productive layer and thus on the light conditions.

They then provided the first estimate of a critical mixing depth:

To get a rough calculation of the possible effect of the turbulence on the phytoplankton, we suppose that a water column of 50 m depth is in continuous vertical circulation, and that the point of compensation is found at a depth of 10 m. The phytoplankton then will be suspended within the illuminated zone only for one fifth of its life time, and only during part of this time the conditions for photosynthesis will be optimal. If they are just optimal at the surface, the average photosynthesis of each cell will be only one tenth of the full value of optimal conditions, that is if the cell has been continuously at the surface. As a rule the light conditions may be optimal one or two metres below the surface, and therefore, the value 1/10 will be too low, and the correct value in our case between 1/5 and 1/10.

The question, whether the phytoplankton during its stay in the lighted zone can accumulate sufficient energy for its further growth, must therefore depend upon the relation between its photosynthesis and respiration. In our example, if the respiration is more than 1/8 of the assimilation, the propagation will stop, and the population will soon be consumed by the zooplankton.

**Figure 1:**
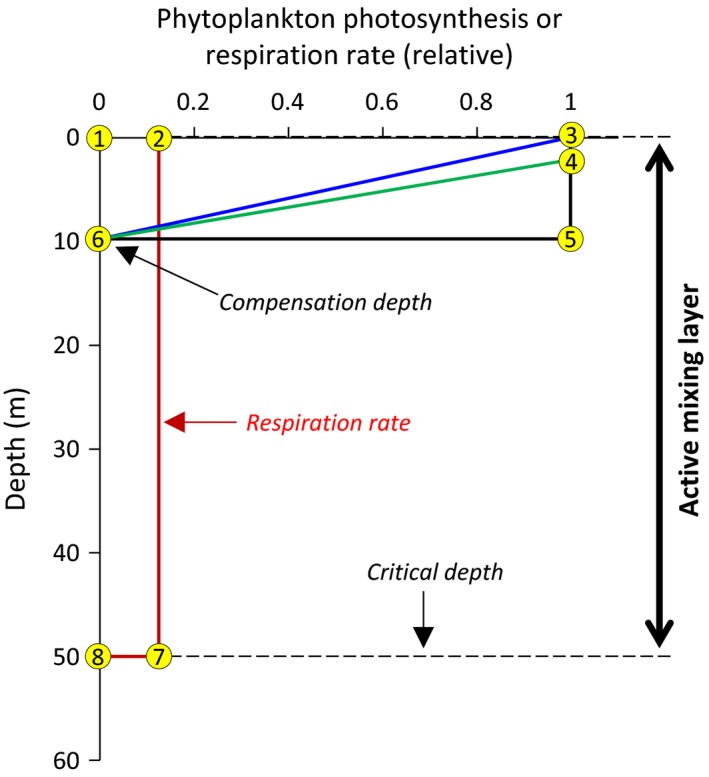
Visual illustration of the original critical depth calculation by Gran and Braarud (1935) for a water column with a surface mixed layer of 50 m. Red line = phytoplankton respiration rate. Black line = Phytoplankton photosynthesis for the initial assumption of constant values to the compensation depth of 10 m. Blue line = Phytoplankton photosynthesis assuming a linear decrease from the surface to the compensation depth. Green line = Phytoplankton photosynthesis assuming a near‐surface layer of light saturation followed by linear decrease to the compensation depth. Yellow numbered circles are used in the text to discuss the calculations

To rephrase the above assessment, consider a water column with an actively mixed surface layer of 50 m depth and where photosynthesis is uniform from the surface to a compensation depth of 10 m (Figure 1). If this photosynthesis (black rectangle defined by points 1, 3, 5, and 6 in Figure 1) is evenly distributed among the mixed layer phytoplankton, then each cell will be growing at one‐fifth of the maximum rate (i.e., at any point in time, only one‐fifth of the population is photosynthesizing). Gran and Braarud recognized that photosynthesis is not uniform, but decreases, from the surface to the compensation depth. They account for this effect by assuming a linear decrease in photosynthesis with depth (blue line in Figure 1) (obviously this is a “first approximation” and is better described by an exponential function). The revised assessment of photosynthesis is thus represented by the area of the triangle in Figure 1 defined by points 1, 3, and 6, which is half the area of the rectangle defined by points 1, 3, 5, and 6. This adjustment causes the photosynthesis of each cell to be one‐tenth rather than one‐fifth of the maximum rate. Gran and Braarud then note that photosynthesis does not, in fact, decrease immediately below the surface, but instead is constant for some depth (in their case, a meter or two) before decreasing. This consideration is illustrated in Figure 1 by the green line, making water column photosynthesis equal to the area defined by the points 1, 3, 4, and 6. Accordingly, each phytoplankton in this water column will have a photosynthetic rate slightly greater than one‐tenth but less than one‐fifth of the maximum rate. If we take the actual value for this photosynthetic rate to be one‐eighth of the maximum rate and assume this value to be equal to the respiration rate of each cell (red rectangle 1, 2, 7, and 8 in Figure 1), then the critical mixing depth for this water column is 50 m.

From their observations and calculations, Gran and Braarud remarked on the spring and summer paucity of phytoplankton in the Bay of Fundy that:

The only possible explanation of this seems to be that the phytoplankton is prevented by the turbulence from accumulating in the illuminated zone. As this zone is shallower than in the gulf of Maine, because of the weaker illumination and the turbidity of the waters, the photosynthesis of the plankton algae, moving up and down with the turbulent waters, may be insufficient to counterbalance their respiration or to give a surplus sufficient to cover the consumption by animals.

In essence, Gran and Braarud envisioned a condition where phytoplankton growth is impossible because the combination of incident light (*I*_0_), attenuation rate (*K*_d_), and turbulent mixing yields an average daily light exposure for mixed layer phytoplankton that was insufficient for gross photosynthesis (GP*) to exceed phytoplankton respiration ($R_{\mathrm{phyto}}^{*}$ ). Only after changes in *I*_0_, *K*_d_, and mixing cause daily light exposure to surpass the critical threshold where GP* = $R_{\mathrm{phyto}}^{*}$ could phytoplankton biomass begin increasing (“blooming”). Thus, the original Critical Depth Hypothesis simply defines when a bloom can (GP* > $R_{\mathrm{phyto}}^{*}$ ) or cannot happen (GP* ≤ $R_{\mathrm{phyto}}^{*}$ ), not when it actually does happen. Gran and Braarud clearly understood that an increase in biomass requires both GP* > $R_{\mathrm{phyto}}^{*}$ and the excess production to exceed grazing (and other) losses (*l*), but these losses were not a component of the critical depth assessment because they were not viewed as dependent on mixed layer light levels.

A key insight of Gran and Braarud was that phytoplankton concentrations have the potential to increase in a mixed layer that is significantly deeper than the compensation depth. What they did not understand was whether or when mixed layer light levels ever became low enough to prevent net growth. This assessment would require some quantitative modeling.

## 5 RILEY'S GEORGES BANK

Gordon Riley published a sequence of manuscripts between 1941 and 1946 regarding the plankton of Georges Bank. In the earliest of these publications, Riley reports that observed changes in phytoplankton biomass were consistent with “the reasoning of Gran and Braarud … that the balance between the effects of turbulence and the increasing vernal radiation initiated the spring flowering” (Riley, 1941) and that the “The rate of increase in [the phytoplankton] population is a function of the ratio of the quantity of organisms in the euphotic zone to the total population [within the mixed layer]” (Riley, 1942). These ideas were further developed mathematically beginning with the relationship (Riley, 1942, equation 1):

(4) $$r=r_{P}\;\frac{Dr_{P}}{Dr_{c}}-r_{c}.$$

Equation (3) retains Riley's original notation where *r* is the specific rate of change in phytoplankton concentration, *r*_P_ is the average gross photosynthetic rate in the euphotic zone, *r*_c_ is the phytoplankton respiration rate, and *Dr*_P_ and *Dr*_c_ are the euphotic depth and mixed layer depth, respectively. Riley then noted that, by assuming *r*_P_, *r*_c_, and *Dr*_P_ to be constant, *r* becomes linearly dependent on 1/*Dr*_c_ and that this relationship was consistent with his March and April field data (*r* = 0.919; see his figure 29), concluding:

It is near enough to a linear form to leave little doubt of the essential correctness of the original proposition [of Gran and Braarud].

Importantly, Riley's use of Equation (3) to evaluate Gran and Braarud's concept reaffirms our statement above that the original Critical Depth Hypothesis concerned only the balance between GP* and $R_{\mathrm{phyto}}^{*}$ and did not enfold other phytoplankton loss processes.

Following his analysis of the March–April data, Riley (1942) turned his attention to the broader seasonal cycle of phytoplankton and the inadequacies of his initial assumptions:

The whole validity of the previous section was based on the assumption that all other factors except r and Dr_c_ remained reasonably constant … The assumption was based on somewhat inadequate experimental evidence … The evidence is of about equal validity that these factors do not remain constant throughout the spring months. … All these things can affect the slope of the plankton–stability relationship, and the real problem therefore is to determine which are the key factors at any one time.

Accordingly, he expands Equation (3) to include phytoplankton losses (*r*_s_) to sinking and grazing:

(5) $$r=\left(r_{P}\;\frac{Dr_{P}}{Dr_{c}}-r_{c}\right)-r_{s},$$

but initially suggests that grazing losses “could hardly have any influence” on the early spring bloom initiation. He abandoned this opinion shortly thereafter.

In 1946, Riley published a synthesis on Georges Bank phytoplankton populations that not only provided a far more sophisticated treatment of plankton annual cycles, but also created the foundation of modern ocean ecosystem modeling. For the current tutorial, however, we will limit our discussion to the most salient points. In recounting his development of thinking regarding Georges Bank plankton populations, Riley notes:

In the first of these publications it was noted that part of the variation that occurred in the distribution of phytoplankton from one part of the bank to another and from one month to the next could be correlated with such factors as the depth of water, temperature and dissolved phosphate and nitrate. Since that time the study of the zooplankton collections has been completed and examination of the data has shown that grazing by zooplankton is important in controlling the size of the phytoplankton population.

Insightfully, he also remarks on the potential timescale of coupling between phytoplankton and zooplankton stocks:

The phytoplankton‐zooplankton relationship has been discussed in some detail in a previous paper…. It was concluded that the predominately negative relationship was due to grazing. The quantities of animals and plants were such as to indicate that the observed relationship could have been established in a very short time, possibly in a day or in a few days

In other words, the difference between phytoplankton division and accumulation rates is primarily due to grazing losses and this relationship is coupled on the time scale of days.

**Figure 2:**
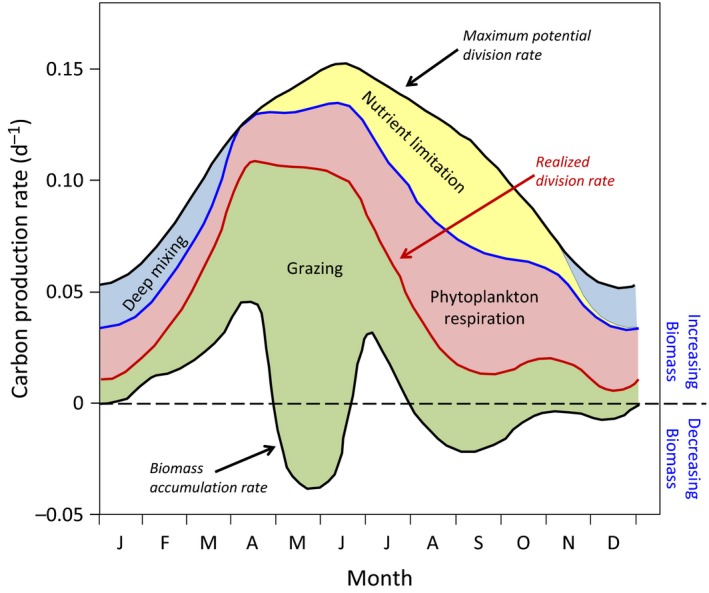
Annual cycles of phytoplankton (top black line) maximum division rate for a nutrient‐replete euphotic zone and (bottom black line) biomass accumulation rate, reproduced from figures 18 and 20 of Riley (1946). Maximum division rates are based on measured incident sunlight and diffuse attenuation. These values are then adjusted downward to account for nutrient stress (yellow shading) and mixing deeper than the euphotic zone (blue shading) to yield gross production rates (blue line). Net production rates (red line) are calculated from gross production by subtracting values of respiration (red shading) calculated as an exponential function of temperature. Finally, biomass accumulation rates are calculated from net production by subtracting grazing losses (green shading) based on observed zooplankton abundances. Biomass accumulation rates above the horizontal black‐dashed line correspond to increasing phytoplankton concentrations, while below the dashed line biomass is decreasing

Riley (1946) begins his theoretical analysis of annual plankton cycles by calculating specific rates of nutrient‐replete gross photosynthesis for the euphotic zone (defined as the 0.0015 g cal cm^−2^ min^−1^ isolume depth)^1^ (upper black line in Figure 2). These values were adjusted downward first to account for nutrient limitation (based on field phosphate records) in summer and autumn (yellow shading in Figure 2) and then to account as a function of *Dr*_p_/*Dr*_c_ (as in Eq. (3)) for deep mixing in winter and spring (blue shading in Figure 2). Respiration rates (red shading in Figure 2) were then subtracted from these adjusted gross photosynthesis rates (blue line in Figure 2) to determine phytoplankton division rates (μ) (red line in Figure 2). What Riley's model showed is that mixing was never deep enough to create a condition of GP* < $R_{\mathrm{phyto}}^{*}$ . In other words, the “critical depth” was never crossed and thus the potential existed throughout the year for phytoplankton biomass to increase. Instead, the primary determinant of whether phytoplankton biomass increased or decreased in the model was zooplankton grazing (green shading in Figure 2), which was described using field zooplankton abundance data and assuming a constant grazing efficiency.

## 6 SVERDRUP REVISITS THE CRITICAL DEPTH HYPOTHESIS

It is not clear to us why the Critical Depth Hypothesis is overwhelmingly accredited in the modern literature to Harald Sverdrup, since the concept was nearly 20 years old by the time of his 1953 publication and in the previous decade Riley provided mathematical evaluations of Gran and Braarud's original concept. What Sverdrup's publication does represent, however, is one of the later treatments where the critical depth still represented the balancing point between GP* and $R_{\mathrm{phyto}}^{*}$, and then other loss processes were accommodated outside of this fundamental threshold. In his second paragraph, Sverdrup (1953) articulates Gran and Braarud's concept as:

The condition for an increase of the total population is that the total production P must exceed the total destruction by respiration, R … This implies that there must exist a critical depth such that blooming can occur only if the depth of the mixed layer is less than the critical value.

This statement, by the way, is where the term “critical depth” was coined. It is also important to note that the term, *R*, in this statement refers specifically to phytoplankton respiration and, as we explain below, the phrase “can occur” does not mean “will occur.”

Sverdrup provided an equation for calculating the critical mixing depth (*D*_cr_) for any given location and time given information on incident sunlight, water column diffuse attenuation (*k*), and the compensation irradiance (*I*_c_):

(6) $$\frac{D_{\mathrm{cr}}}{1-e^{-kD_{\mathrm{cr}}}}=\frac{1}{k}\frac{I_{o}^{*}}{I_{c}}$$

where we have again retained the original notation and *I*_o_* is the 24 hr average incident total solar energy corrected for both surface reflection and the fraction available for photosynthesis. Equation (5) was further simplified (i.e., by assuming that $1-e^{-kD_{\mathrm{cr}}}$ ~ 1) to:

(7) $$\frac{D_{\mathrm{cr}}}{D_{c}}=\frac{e^{kD_{c}}}{kD_{c}},$$

where *D*_c_ is the compensation depth when subsurface irradiance equals *I*_c_.

**Figure 3:**
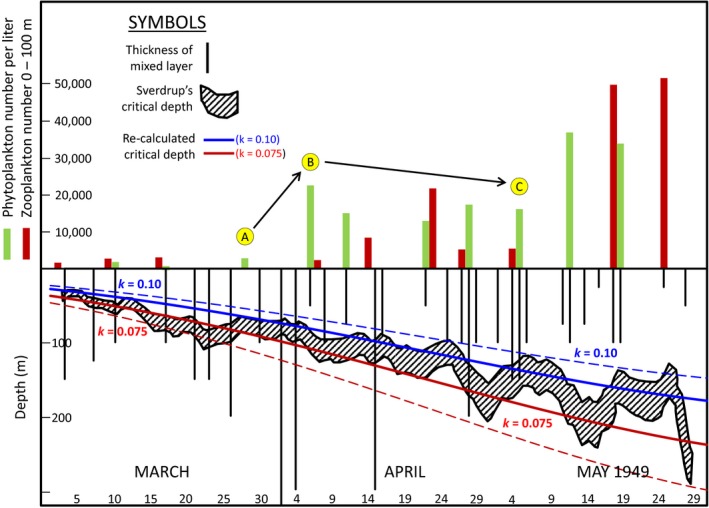
“Digital age” reproduction of figure 2 of Sverdrup (1953). Top half of figure shows phytoplankton (green bars) and zooplankton (red bars) abundances observed between March and May 1949 at Weather Station “M.” Bottom half of figure shows mixed layer depths (vertical black lines) and the range of Sverdrup's critical depth estimates (hatched area) for diffuse attenuation coefficients (*k*) of 0.10 and 0.075 m^−1^. Using these values for *k* and assuming constant cloudiness values following Sverdrup's scale, our recalculated critical depth values are shown by the solid red (*k* = 0.075 m^−1^; cloudiness = 7.5) and blue lines (*k* = 0.10 m^−1^; cloudiness = 7.5) and dashed red (*k* = 0.075 m^−1^; cloudiness = 6.0) and blue lines (*k* = 0.10 m^−1^; cloudiness = 8.5). For these calculations, we assumed a constant respiration rate of 0.14 g cal cm^−2^ hr^−1^, which is the average of two values given by Sverdrup for the studies of Jenkins (1937; 0.13 g cal cm^−2^ hr^−1^) and Pettersson, Höglund, and Landberg (1934; 0.15 g cal cm^−2^ hr^−1^)

Sverdrup applied Equation (1) to field observations at Weather Station “M” (66°N, 2°E) to evaluate the relationship between calculated critical depth values and observed changes in phytoplankton abundance between March and May 1949. Figure 3 is a “digital age” reproduction of Sverdrup's Figure 2, with a few of our own additions. In the lower half of the figure, Sverdrup's *D*_cr_ time series is shown by the hatched area, with the range in values corresponding to *k* assignments between 0.075 and 0.10 m^−1^. Our own recalculation of this time series is shown by the blue (*k *=* *0.10 m^−1^) and red (*k *=* *0.075 m^−1^) lines, where we have assumed a value for $R_{\mathrm{phyto}}^{*}$ of 0.14 g cal cm^−2^ hr^−1^ (i.e., the average of the two phytoplankton respiration rates cited by Sverdrup for a pure culture of *Coscinodiscus excentricus* and a mixed natural population from Gullmar Fjord, Sweden). The “ragged” nature of Sverdrup's time series compared to our calculated values is due to his original assessment including information on local cloud conditions, whereas we assumed a constant cloudiness (i.e., a value of 7.5 on Sverdrup's scale). The entire range of original *D*_cr_ values is captured by slightly changing our chosen cloudiness value upward (dashed blue line in Figure 3; cloudiness = 8.5) and downward (red‐dashed line in Figure 3; cloudiness = 6.0). Thus, no additional phytoplankton loss processes need to be included to reproduce Sverdrup's critical depth values.

The vertical black lines in the lower half of Figure 3 indicate measured mixed layer depths, whereas the green bars in the top half indicate phytoplankton concentrations. Sverdrup noted that the first significant rise in phytoplankton number (i.e., from the yellow circle “A” to circle “B”) corresponded to a transition in mixing from deeper than *D*_cr_ to shallower than *D*_cr_. However, from this point forward, phytoplankton concentration changes showed no dependence on the relationship between mixing depth and *D*_cr_ (e.g., mixed layer depths are generally shallower than *D*_cr_ from the yellow circle “B” to circle “C,” yet phytoplankton concentrations are overall on the decline). Sverdrup attributes this result to zooplankton grazing:

During the remaining part of April and the first half of May, when the depth of the mixed layer was only moderately smaller than the critical depth, the phytoplankton population remained moderately large, and did not increase systematically. At the same time copepods appeared in greater numbers, indicating that considerable grazing took place.

and he reports zooplankton abundances (red bars in top half of Figure 3) to qualitatively support this argument. Thus, the critical depth is a threshold defining when phytoplankton can and cannot divide. When mixed layer light levels exceed this threshold, phytoplankton biomass will only increase if, in the words of Gran and Braarud (1935), this “surplus [is] sufficient to cover the consumption by animals,” to which we add “and other loss processes.”

## 7 METAMORPHOSIS

As graduate students, the Critical Depth Hypothesis was taught to many of us in a manner inconsistent with its original conception. Specifically, the “respiration” term was redefined from that of the phytoplankton to that of all loss processes (e.g., Platt, Bird, & Sathyendranath, 1991; Smetacek & Passow, 1990). This revised interpretation is pervasive in today's literature, yet we have not identified a specific publication to cite as its origin. Perhaps, a precursor to this change can be found in a statement by Sverdrup:

The compensation depth is defined as the depth at which the energy intensity is such that the production by photosynthesis balances destruction by respiration. This energy level … must, for instance, lie higher for a mixed population of phytoplankton and zooplankton than for a pure phytoplankton population.

What Sverdrup was alluding to here is that the “phytoplankton compensation point” can be considerably lower than the “community compensation point.” Riley (1957) subsequently investigated variability in the community compensation point of natural plankton assemblages, but neither he nor Sverdrup suggested treating this community‐level balancing point in the same manner as the GP* vs. $R_{\mathrm{phyto}}^{*}$ balancing point of the original Critical Depth Hypothesis.

So, why did the interpretation of the Critical Depth Hypothesis go through this metamorphosis? We cannot be certain, but perhaps the simple reason is that in its original form it did not provide the desired prediction of when a bloom will happen, only when it can or cannot happen. By reinterpreting “respiration” as all losses, the Critical Depth Hypothesis is transformed into a prediction of when phytoplankton biomass actually begins increasing. While this may seem like a minor detail, it actually represents a major deviation because it implies that the balance between phytoplankton division and loss rates is predictable from information on the mixed layer light environment. Gran and Braarud (1935) and Sverdrup (1953) clearly recognized that grazing and other losses play a crucial role in determining whether phytoplankton biomass increases or decreases, but they did not suggest that these losses could be related to mixed layer light. Instead, they restricted their definition of respiration in their calculations to that of the phytoplankton, which they assumed to be sufficiently constrained to treat as a constant. As discussed below, the modern reassignment of respiration to all losses creates a model with either no predictive capabilities or untenable conclusions regarding biomass dynamics over the annual cycle.

## 8 A PROPOSAL

We have come to a point in our tutorial where it is necessary to introduce some new terminology; otherwise, our remaining discussion will become hopelessly confusing. We also propose that this terminology be adopted in all future publications on blooms.

Many recent studies (including some of our own) have claimed to test, refute, or confirm “Sverdrup's Critical Depth Hypothesis.” We need to stop using this phrase. The first issue, as noted above, is that the concept has two fundamentally different forms that are differentiated by their interpretation of “respiration.” The second issue is that both forms address the average light level experienced by phytoplankton in the mixed layer, which is a function not just of mixing depth, but also incident sunlight and the diffuse attenuation coefficient. The essence of these two hypotheses resides in the balancing points they are addressing. The hypothesis of Gran and Braarud (1935) focuses on the relationship between GP* and $R_{\mathrm{phyto}}^{*}$, so we propose the phrase “Gran and Braarud's Critical Photosynthesis Hypothesis.” The second hypothesis focuses on the balance between phytoplankton division and loss rates, so we propose the “Critical Division Rate Hypothesis,” which could perhaps be cited as Smetacek and Passow (1990) although this would be a disservice since these authors were articulating how the concept has little value in understanding phytoplankton annual cycles.

The next issue to deal with is the word “Critical.” Neither of the above two hypotheses should be cited as originating the concept that phytoplankton blooms are initiated by improved mixed layer light conditions. More appropriate references for this might be Gran (1932) and Atkins (1932) although many earlier studies contributed to the idea (Mills, 2012). Rather, the significance of the term “Critical” is that it denotes a threshold. For *Gran and Braarud's Critical Photosynthesis Hypothesis*, it refers to a critical light level below which phytoplankton division is arrested. For the *Critical Division Rate Hypothesis*, it refers to a critical division rate below which phytoplankton biomass decreases (i.e., *r* is negative because μ < *l*; Equation (1)). Thus, the utility and even the validity of these hypotheses hinge on these critical thresholds.

## 9 EVALUATING GRAN AND BRAARUD'S CRITICAL PHOTOSYNTHESIS HYPOTHESIS

There is no question that mixed layer light conditions in the natural world can be sufficiently low that phytoplankton division is arrested. Those conditions are called polar night. The important question instead is how often and where phytoplankton division in the mixed layer is prohibited by light. The answer to this question requires an appropriate estimate of the compensation irradiance and thus of respiration, which was an issue of concern early‐on:

The data published as yet on the respiration of the plankton algae are not plentiful. … The few observations yet available are of course quite insufficient for drawing general conclusions, but they are sufficient to show that the daily respiration of the phytoplankton is relatively high compared with its photosynthesis. (Gran & Braarud, 1935)

The few available measurements of the respiration of pure diatom cultures have not yielded precise results. Observed rates have varied from one species to another as well as during different stages of growth of the same culture. The recorded values differ by a factor of 10 to 20, and there are not enough of them to draw a good average. (Riley, 1946)

To put a “finer point” on the respiration problem, what we need is a value representative of phytoplankton populations that are fully acclimated to near‐compensation light levels (Lindemann, Backhaus, & St John, 2015). In addition, it is desirable to have such a number for the types of phytoplankton species that dominate under high‐latitude, deeply mixing winter conditions. To our knowledge, measurements satisfying both of these desires have not been made, but we do have some useful constraints.

Smetacek and Passow (1990) discussed in some detail important considerations regarding the respiration problem. In particular, it is now well recognized that respiration rate decreases with decreasing growth rate (Geider, 1992; Geider, MacIntyre, & Kana, 1998; Halsey, Milligan, & Behrenfeld, 2010; Laws & Bannister, 1980; Lindemann et al., 2015; Penning De Vries, Brunsting, & van Laar, 1974), and thus with decreasing light. This is why an evaluation of *Gran and Braarud's Critical Photosynthesis Hypothesis* requires respiration values from phytoplankton acclimated to very low light. Geider, Osborne, and Raven (1986) conducted such an experiment with the temperate diatom *Phaeodactylum tricornutum*, where cultures were grown at four extremely low light levels ranging from 0.065 to 0.302 mole quanta m^−2^ day^−1^ (Table 2). A strong linear relationship between growth rate and irradiance is observed in these data (μ = 0.18 irradiance − 0.008; r^2^ = 0.97) that, when solved for a value of μ = 0, yields a compensation irradiance of 0.044 mole quanta m^−2^ d^−1^. This value is roughly a factor of 10 lower than the compensation irradiance assumed by Sverdrup (1953) (~0.6 mole quanta m^−2^ day^−1^). *P. tricornutum*, unfortunately, is not a dominant species in low‐light natural environments (e.g., Backhaus et al., 2003; Dale, Rey, & Heimdal, 1999; Halldal, 1953) and its compensation irradiance likely overestimates that of high‐latitude winter populations.

**Table 2:** Division rate of the temperate diatom *Phaeodactylum tricornutum* at very low light levels as reported by Geider et al. (1986). Grow irradiance is shown both as an incident flux and daily dose

Irradiance (μmole m^−2^ s^−1^)	Irradiance (mole m^−2^ day^−1^)	Specific division rate (day^−1^)
0.75	0.065	0
1.3	0.112	0.016
2.1	0.181	0.026
3.5	0.302	0.045

**Figure 4:**
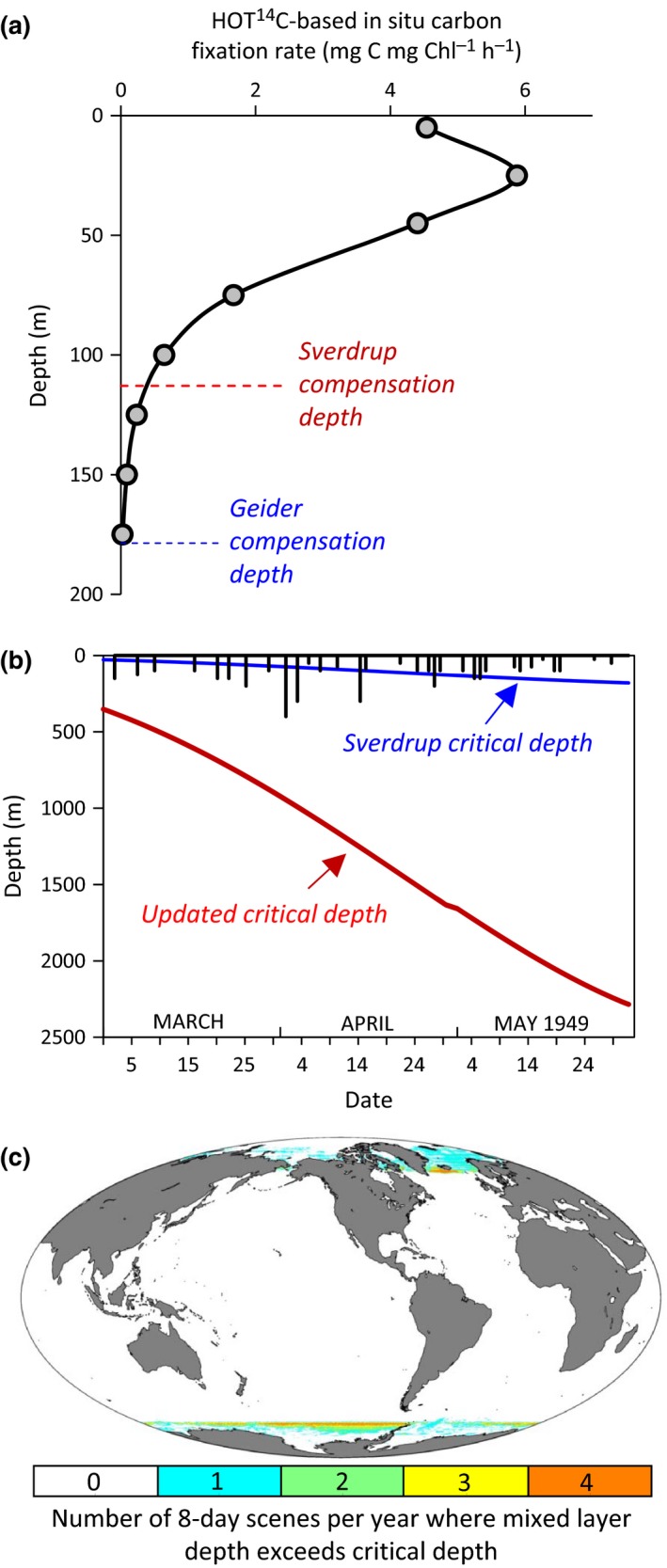
Modern compensation depths and critical depths. (a) Productivity and compensation depths for an example stratified water column measured at the Hawaii Ocean Time‐series (HOT) site. Black line, gray symbols = ^14^C uptake rates−dark‐bottle values for samples from the surface to 175 m. Blue‐dashed line = compensation depth based on Geider et al. (1986) where daily irradiance is 0.044 mole quanta m^−2^ day^−1^ (see text). Red‐dashed line = compensation depth based on Sverdrup (1953) where daily irradiance is ~0.6 mole quanta m^−2^ day^−1^. (b) Updated critical depth values for Weather Station “M” from March through May 1949. Vertical black lines = mixed layer depths reported by Sverdrup (1953). Blue line = critical depth values for Sverdrup's compensation irradiance of ~0.6 mole quanta m^−2^ day^−1^, a diffuse attenuation coefficients of 0.10 m^−1^, and a cloudiness index = 7.5 (same as solid blue line in figure 3). Red line = critical depth values calculated using a compensation irradiance of 0.044 mole quanta m^−2^ day^−1^ (Fig. 4a, see text), a diffuse attenuation coefficient of 0.10 m^−1^, and a cloudiness index = 7.5. (c) Global assessment of the number of 8‐day scenes per year when mixed layer depths (taken as the depth where density is 0.030 kg/m^3^ greater than the value at 10 m) exceed the critical mixing depth (based on a compensation irradiance of 0.044 mole quanta m^−2^ day^−1^). Values in polar regions exclude days of polar night since mixing depth is irrelevant to the ability of phytoplankton biomass to increase. Results are average annual values for the period 2003–2015 and based on 8‐day MODIS Aqua diffuse attenuation coefficients and cloud‐corrected incident photosynthetically available radiation and mixed layer depths from the HYCOM model (source: https://hycom.org/). To extend satellite fields beyond the limit of seasonal coverage at high latitudes, we assume values for PAR of 1 mole quanta m^−2^ day^−1^ and diffuse attenuation coefficients for PAR of 0.045 m^−1^

A second approximation for the needed compensation irradiance can be derived from field ^14^C uptake measurements. The benefit here is that, in a permanently stratified water column, phytoplankton populations near the compensation depth have been selected for low‐light adapted species, albeit not the same species as found in winter at high latitudes. The drawback of ^14^C data is that the measurement always yields positive values even when photosynthesis is arrested. This issue can be partially addressed by subtracting parallel ^14^C uptake values measured in dark bottles. Such data are shown in Figure 4a, which gives a vertical profile of in situ daily ^14^C uptake corrected for dark bottle uptake as measured at the Hawaii Ocean Time‐series (HOT) site. These data indicate significant (*p *> .05) net production to a depth of 175 m, or a light level of ~0.047 mole quanta m^−2^ day^−1^. Reassuringly, this compensation irradiance is very near that estimated above from the Geider et al. (1986) data (Figure 4a).

With our revised compensation values, we can now revisit Sverdrup's time series at Weather Station “M.” In Figure 4b, Sverdrup's mixed layer depths are again shown as the vertical black lines and his critical depths for net photosynthesis are shown by the blue line (same as solid blue line in Figure 3). The solid red line in this figure shows the critical depths for our compensation irradiance of 0.044 mole quanta m^−2^ day^−1^ and a diffuse attenuation coefficient of 0.10 m^−1^. These data indicate that mixing depths were far from sufficient to cause GP* > $R_{\mathrm{phyto}}^{*}$ throughout the Weather Station “M” study. In other words, Sverdrup's observations provided no support for *Gran and Braarud's Critical Photosynthesis Hypothesis*.

We now return to the original question posed in this section. Satellite ocean color data provide 8‐day resolution global fields of diffuse attenuation coefficients and cloud‐corrected incident photosynthetically available radiation (PAR). Equivalent resolution global fields of mixed layer depth are also available using models tuned to available in situ observations (e.g., http://www.science.oregonstate.edu/ocean.productivity/). With these data, we calculated the number of 8‐day average scenes per year (excluding polar night) for each 9 km satellite pixel where mixed layer average light levels are less than the compensation irradiance of 0.044 mole quanta m^−2^ day^−1^. What we find is that, aside from a few 8‐day periods at high latitudes near polar night, mixed layer light levels are at all times and everywhere sufficient for phytoplankton growth (Figure 4c). More specifically, only 6% of the total spatial pixels have any instances where GP* < $R_{\mathrm{phyto}}^{*}$ and, of these, the condition is found for 2 or less 8‐day periods in 60% of all cases. This assessment is likely also conservative because we have assumed uniform mixing to the bottom of a density‐defined mixed layer (taken here as a density change of 0.030 kg/m^3^ from the value at 10 m [de Boyer Montégut, Madec, Fischer, Lazar, & Iudicone, 2004]), which often significantly overestimates active turbulent mixing (Franks, 2014).

The take‐home message of this section is that *Gran and Braarud's Critical Photosynthesis Hypothesis* has little relevance to annual cycles of phytoplankton biomass in the open ocean. The one exception is very near and during polar night and an excellent field‐based example of such conditions was provided by Venables, Clarke, and Meredith (2013). In their study, phytoplankton accumulation rates (*r*) were measured over two complete annual cycles in the Southern Ocean. During both years, blooming (positive *r*) began ~2 weeks after polar night ended. Since these accumulation rates include all loss processes, this finding implies that mixed layer light levels were adequate for GP* > $R_{\mathrm{phyto}}^{*}$ even earlier.

## 10 THE CRITICAL DIVISION RATE HYPOTHESIS

In this section, we use a numerical modeling exercise to evaluate the *Critical Division Rate Hypothesis*, but before starting that let us revisit two earlier points. First, the fundamental tenet of the *Critical Division Rate Hypothesis* is that there exists a threshold phytoplankton division rate below which biomass decreases and above which biomass increases. Second, a prerequisite for any valid bloom hypothesis is that it must not result in untenable conclusions regarding biomass dynamics at other times of the year. Thus, application of the *Critical Division Rate Hypothesis* should not only explain when blooming begins, but also yield an annual cycle in *C*_phyto_ that is consistent with observations. Okay, we are now ready to do some modeling.

**Figure 5:**
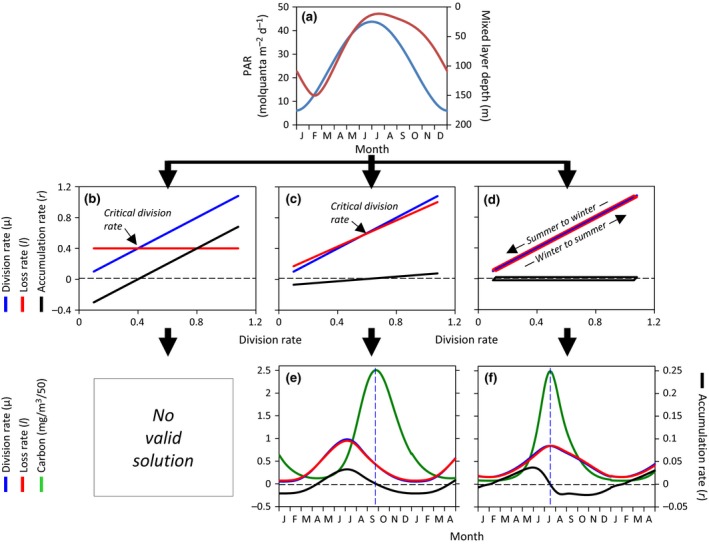
Illustration of contrasting bloom hypotheses. (a) Annual cycles in (blue line) incident photosynthetically active radiation (PAR) and (red line) mixed layer depth representative of a high latitude, open ocean bloom forming region. (b–d) Phytoplankton division, loss, and accumulation rates plotted as a function of division rate for contrasting bloom concepts. (b) Critical Division Rate Hypothesis where loss rates are assumed constant. Note that there are no parameterizations of this concept that yield valid annual cycles in phytoplankton biomass when applied to the PAR and MLD data in panel (a). (c) Critical Division Rate Hypothesis where loss rates are assumed to covary with division rates, but a threshold exists (black arrow) that must be surpassed for phytoplankton biomass to increase. Accordingly, biomass decreases at all division rates below this threshold. (d) Conceptual model of plankton ecosystems where changes in phytoplankton loss rates are temporally lagged behind changes in division rate. (e, f) Phytoplankton carbon concentrations and division, loss, and accumulation rates associated with the conceptual relationships shown in (c) and (d), respectively, when applied to the annual cycles in PAR and MLD shown in panel (a). Note *x*‐axis extends for 16 months. For these calculations, division rate was assumed to be a linear function of median mixed layer PAR. Horizontal dashed black line in (b)–(f) corresponds to a biomass accumulation rate = 0. Vertical dashed blue line in (e) and (f) corresponds to the climax in biomass and provides a reference against which maxima in the three biologic rates can be compared. See Appendix S1 for mathematical formulation for the three scenarios shown panels b, c, and d

The simplest formulation of the *Critical Division Rate Hypothesis*, and one frequently adopted in the recent literature, is where the sum of all phytoplankton loss rates (*l*) is assumed constant (Figure 5b, red line). With this assumption, the rate of change in phytoplankton biomass (*r*) varies in a one‐to‐one manner with division rate (μ) and *r* is negative (i.e., biomass is decreasing) below the critical division rate (black arrow in Figure 5b) and positive (i.e., biomass is increasing) above it. If we now apply this conceptual model to representative annual cycles of incident surface photosynthetically active radiation (PAR) and mixed layer depths (MLD) in the subpolar North Atlantic (Figure 5a) where a long history of bloom studies have been conducted, we find that it is impossible to recreate a valid annual cycle in phytoplankton concentration (see Appendix S1 for modeling details). Any assigned value for *l* that sustains, for example, realistic phytoplankton biomass through winter (e.g., ~2 mg C m^−3^) subsequently yields biomass values that are far too high at the bloom climax (i.e., >5,000 mg C m^−3^). Conversely, any value for *l* that gives reasonable climax values (e.g., ~250 mg C m^−3^) necessarily decimates the phytoplankton population during winter (i.e., <0.005 mg C m^−3^). Clearly, this conception of the *Critical Division Rate Hypothesis* is unsound and should neither be taught to students, implemented in models, nor promoted in the literature.

Obviously, few plankton ecologists would suggest that phytoplankton‐specific loss rates are constant in time. We can therefore revise our formulation to the other extreme where loss rates closely parallel phytoplankton division rates (Figure 5c). To retain the tenet of a threshold division rate (black arrow in Figure 5c), this formulation of the *Critical Division Rate Hypothesis* requires that values of *l* (Figure 5c, red line) are slightly higher than μ (Figure 5c, blue line) at low division rates and slightly lower than μ at high division rates. By carefully selecting a slope and intercept for this division–loss relationship (i.e., the model is extremely unstable, Appendix S1) and then applying it to our annual cycles in PAR and MLD (Figure 5a), this version of the *Critical Division Rate Hypothesis* can produce an annual cycle in phytoplankton biomass with appropriate minima and maxima (Figure 5e). However, the timing of this cycle is incorrect, with a spring minimum (~April) and an autumn climax (~September). The reason for this shift is that the requirement for a “critical” division rate necessitates that biomass continues to decrease in the spring until the threshold is met and that biomass continues to increase through the summer until division rate once again falls below the threshold. Additional constraints, such as mid‐summer nutrient depletion, may be added to this model to shift the timing of the climax, but sustained reductions in biomass well into spring remain an unavoidable consequence of assuming a threshold division rate.

The take‐home message of this section is that formulations of the *Critical Division Rate Hypothesis* cannot reproduce annual cycles in phytoplankton biomass that are consistent with field observations. More importantly, the assumption of a unique balancing point between phytoplankton division and loss rates is wholly inconsistent with our understanding of zooplankton ecology (Smetacek & Passow, 1990). Finally, recent field and satellite observations have shown that the value of μ does not determine the sign of *r* (e.g., Behrenfeld, 2010, 2014; Behrenfeld et al., 2016, 2017; Boss & Behrenfeld, 2010). It is time to abandon the *Critical Division Rate Hypothesis* as a useful framework for understanding phytoplankton blooms and annual biomass cycles.

## 11 CRITICAL TURBULENCE SIDEBAR

There have been numerous publications in the recent literature building on the *Critical Turbulence Hypothesis* of Huisman, van Oostveen, and Weissing (1999) (Chiswell, 2011; Huisman, Arrayás, Ebert, & Sommeijer, 2002; Taylor & Ferrari, 2011a, 2011b). These studies focus on the physics of upper ocean turbulence, how it can both give rise to phytoplankton vertical structure in a layer of uniform density and how it can allow for increases in phytoplankton biomass in a water column with an apparently deep mixed layer. The important message of these publications is that the temporal dynamics of phytoplankton biomass is directly dependent on the depth of turbulent mixing, rather than the vertical uniformity of density (Franks, 2014). However, the *Critical Turbulence Hypothesis* still assumes that an increase in biomass requires division rates to cross a “Critical” threshold and thus is conceptually flawed for the same reasons as discussed in the previous section regarding the *Critical Division Rate Hypothesis*.

## 12 THE DISTURBANCE RECOVERY HYPOTHESIS

The *Disturbance Recovery Hypothesis* originated from satellite observations of the Subarctic Atlantic (Behrenfeld, 2010) and has continued to evolve through expanded analyses with field, modeling, and additional satellite data (Behrenfeld & Boss 2014; Behrenfeld, Doney, Lima, Boss, & Siegel, 2013; Behrenfeld, 2014; Behrenfeld et al., 2016, 2017; Boss & Behrenfeld, 2010). It is intended as a basic framework for understanding blooms within the context of complete phytoplankton annual cycles. Its focus is on the interplay between “top down” (i.e., loss processes) and “bottom up” (i.e., resources for cell division) processes that together govern biomass dynamics. Central tenets of the *Disturbance Recovery Hypothesis* are:

1. Planktonic ecosystems have a strong attraction toward equilibrium solutions for phytoplankton biomass (*C*_phyto_; mg C m^−3^). Environmental “disturbances” of various forms allow *C*_phyto_ to change by impacting the balance between μ and *l*. Subsidence of these disturbances promotes a “recovery” in the balance between μ and *l* and thus yields a new equilibrium state for *C*_phyto_ (Behrenfeld & Boss 2014; Behrenfeld et al., 2016, 2017).
2. Equilibrium values for *C*_phyto_ covary with μ, but not in a one‐to‐one manner (e.g., a factor of 10 change in μ may be associated with only a factor of 2 change in *C*_phyto_). This relationship is the consequence of a trophic cascade, where carnivory prevents herbivores from maintaining constant *C*_phyto_ at all values of μ (Behrenfeld & Boss 2014; Behrenfeld et al., 2013; Behrenfeld, 2014; see also Appendix S2).
3. Accumulation rates (*r*) for *C*_phyto_ covary with the specific rate of change in μ, not the absolute value of μ. Thus, for any μ, *r* can have any value <μ and the sign of *r* is dependent on the direction of change in μ. Positive values of *r* result from accelerations in μ, whereas negative values of *r* result from decelerations in μ. Accelerations and decelerations in μ represent one form of “disturbance” and result from “bottom up” factors such as mixed layer light levels and nutrient availability (Behrenfeld, 2014; Behrenfeld et al., 2016, 2017).
4. Physical disturbances (e.g., mixed layer deepening, freshwater input) can have compound impacts on the balance between μ and phytoplankton loss rates (*l*). For example, when mixed layer deepening entrains relatively phytoplankton‐free water from depth, it dilutes the mixed layer population and reduces encounter rates between the phytoplankton and their herbivore grazers and viruses. This effect reduces *l*. Simultaneously, a deepening mixed layer also decreases the daily light exposure of the plankton, which consequently reduces μ. If the “dilution effect” is stronger than the “light effect,” then ∑*C*_phyto_ will increase despite declining μ but *C*_phyto_ will continue to covary with μ (Behrenfeld, 2010; Behrenfeld & Boss 2014; Behrenfeld et al., 2013; Boss & Behrenfeld, 2010).

The *Disturbance Recovery Hypothesis* accounts for the observations that *r* can have similar values in late winter, spring, and early summer in the subarctic Atlantic, that ∑*C*_phyto_ increases in early winter during convective mixed layer deepening, and that the early winter increase in ∑*C*_phyto_ is greater with deeper mixing (Behrenfeld, 2010; Behrenfeld et al., 2013; Boss & Behrenfeld, 2010). It also explains why ∑*C*_phyto_ may or may not increase with winter convection (Llort, Lévy, Sallée, & Tagliabue, 2015). The *Disturbance Recovery Hypothesis* is entirely consistent with early views that springtime improvements in mixed layer light levels promote phytoplankton blooming (Atkins, 1932; Gran, 1932), but it also predicts in a testable manner precisely how this relationship behaves in terms of accelerations and decelerations in μ (Behrenfeld, 2014; Behrenfeld et al., 2016, 2017). These same mechanisms govern *C*_phyto_ variability throughout the year and can account for interannual variations in bloom climax concentrations (Behrenfeld et al., 2016, 2017).

At the heart of the *Disturbance Recovery Hypothesis* is the notion that phytoplankton division and loss rates are perpetually and closely coupled, such that the rate of change in *C*_phyto_ is determined by the rate of change in μ (tenet #3 above). This relationship is due to a temporal lag between μ and *l*. As a way of envisioning this relationship, consider a coupled system where changes in *l* are time lagged behind μ by a period of 1 day. Thus for the time interval (d*t*) from *t*_0_ to *t*_1_, $l_{t_{1}}=\mu_{t_{0}}$ and the rate of change in *C*_phyto_ (*r*_t1_) is:

(8) $$r_{t}1=\mu_{t}1-l_{t}1=\mu_{t}1-\mu_{t0}=\frac{d\mu }{dt}\Delta t$$

making *r* dependent on the rate of change in division (dμ/d*t*) (Behrenfeld et al., 2016, 2017). This relationship between *r* (day^−1^) and dμ/d*t* (day^−2^) exists whether the lag between losses and division is 1 day or longer, but longer lags (weaker coupling) yield larger cycles in *C*_phyto_. In these coupled systems, *l* (Figure 5d, red line) is slightly less than μ (Figure 5d, blue line) when division rates are increasing, such as during the winter‐to‐summer increase in mixed layer light levels in the subarctic Atlantic. Conversely, *l* is slightly greater than μ when division rates are decreasing, such as during the subarctic Atlantic summer‐to‐winter transition (Figure 5d).

Applying the model in Figure 5d to the PAR and mixed layer data in Figure 5a yields the annual cycles shown in Figure 5f. In contrast to results for the *Critical Division Rate Hypothesis* (Figure 5e), the model based on the *Disturbance Recovery Hypothesis* shows an increase in *C*_phyto_ as soon as the mixed layer stops deepening and μ starts to rise (Figure 5f, green and blue lines). We also see that the bloom climax now coincides with the maximum μ. Thus, bloom termination does not require nutrient limitation, only that μ stops accelerating (e.g., mixed layer light stops increasing due to combined changes in mixing depth, incident sunlight, and attenuation [self‐shading, colored dissolved organic matter, etc.]) such that loss rates “catch up.” Finally, the peak in *r* (Figure 5f, black line) corresponds to the most rapid acceleration in μ and occurs well before the maximum in μ (Figure 5f, blue line). All of these outcomes of the *Disturbance Recovery Hypothesis* are consistent with satellite observations (e.g., Behrenfeld, 2010, 2014; Behrenfeld et al., 2013, 2016, 2017), field data (Boss & Behrenfeld, 2010), and even a recent multi‐year Martha's Vineyard time series of *Synechococcus* abundances (Hunter‐Cevera et al., 2016). It is humbling to further note the consistency between the cycles shown in Figure 5f and those divined from scant field data by Riley in 1946 (Figure 2).

## 13 TUTORING

Okay, we have covered a lot of ground now (and a lot of years!) and it is time to synthesize some main points. We will start with a one‐paragraph summary of all the preceding sections. We set out in this tutorial by recognizing annual cycles in phytoplankton biomass as consequences of imbalanced division (μ) and loss (*l*) rates, where a prolonged excess of division over loss yields a bloom. We then noted that the key issue of concern is defining the conditions under which μ* *> *l*, which we refer to as “blooming.” Our foray into the literature of the early 1900s next left us with a benchmark in the works of Gran (1932) and Atkins (1932), where deep winter mixing charges the surface layer with nutrients and subsequent mixed layer shoaling and increasing sunlight first stimulates a rise in *C*_phyto_ and then curtails the bloom by promoting nutrient exhaustion. It is against this benchmark that we then cast subsequent bloom hypotheses. As a terse synopsis, the essential new element of *Gran and Braarud's Critical Photosynthesis Hypothesis* was the addition of a threshold mixed layer light level that must be surpassed for photosynthesis to exceed phytoplankton respiration, thereby permitting but not guarantying blooming (i.e., other losses still have to be accounted for but are not a function of mixed layer light levels). The essential modification of the *Critical Division Rate Hypothesis* and the *Critical Turbulence Hypothesis* was that the threshold mixed layer light level was redefined as distinguishing μ < *l* from μ* *> *l*, thus identifying when blooming actually does happens. Finally, the *Disturbance Recovery Hypothesis* predicts *C*_phyto_ to increase and decrease at a rate proportional to the rate of change in μ and allows physical processes (e.g., deepening mixed layer) to create a condition of μ* *> *l* even when μ is on the decline. We added to our description of bloom hypotheses some simple modeling results (Figure 5) that illustrate the implications of contrasting concepts on derived annual cycles in μ, *l*,* r*, and *C*_phyto_. Now, we would like to suggest some hopefully useful “tools” for your consideration.

When you read through the bloom literature, you will find many instances suggesting support for the “Critical Depth Hypothesis.” In most cases, what the authors really mean is that their data are consistent with increasing mixed layer light levels driving an increase in *C*_phyto_. However, this relationship is common to all of the concepts discussed above, from Gran/Atkins (1932) through to the *Disturbance Recovery Hypothesis*. Evaluating contrasting hypotheses requires instead a focus on the essential elements distinguishing these hypotheses. For example, verifying *Gran and Braarud's Critical Photosynthesis Hypothesis* or the *Critical Division Rate Hypothesis* requires that the envisioned critical thresholds in GP* and μ, respectively, be demonstrated. With respect to the latter hypothesis, confirming a threshold in μ requires observations spanning the full range of phytoplankton division rates (i.e., the full annual cycle). Most field studies are far too short in duration to meet this requirement although there are some splendid exceptions (e.g., Venables et al., 2013).

**Figure 6:**
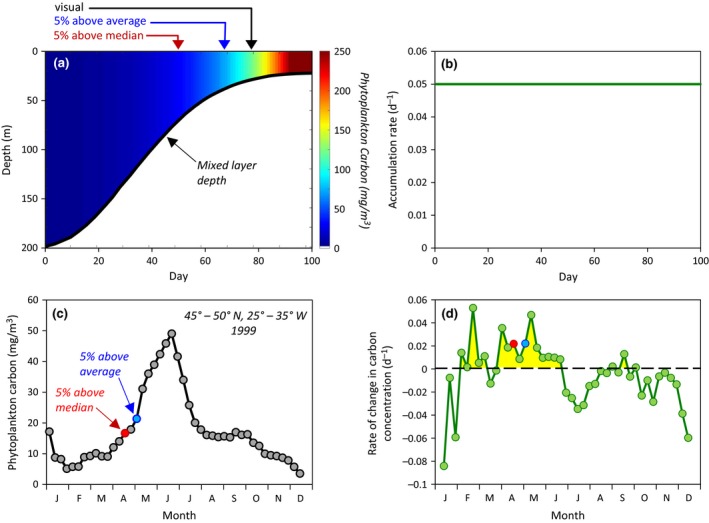
Examples of how untransformed phytoplankton concentration data can mislead interpretations of biomass dynamics and blooms. (a) Vertically resolved phytoplankton biomass modeled over a 100 day period of mixed layer shoaling. Arrows at top identify three different bloom initiation dates, with the black arrow representing a subjective visual date based on a change in color and the blue and red based on the quantitative criterion of biomass exceeding by 5% the average and median concentrations of the time series, respectively. All three of these assessments are wrong, as shown in panel (b) which give the actual biomass accumulation rate assumed in the model (i.e., there was not “initiation” event, simply a constant rate of exponential growth). (c) An 8‐day resolution annual cycle in phytoplankton biomass observed in 1999 for the region 45°–50°N latitude and 25°–35°W longitude (from Behrenfeld, 2014). Here, the blue and red symbols indicate when springtime biomass exceeds by 5% the annual average and median concentrations, respectively. (d) Specific rate of change in carbon concentration calculated from the 1999 satellite record with the blue and red symbols as in (c). Yellow shading indicates times of increasing biomass (i.e., “blooming”). Note that neither the blue nor red symbol demarks any specific event during the bloom

Another major issue with the bloom literature is how data are analyzed and reported. We strongly suggest paying close attention to this one, so here are some key points. As stated above, an understanding of phytoplankton biomass dynamics demands an analysis of biologic rates (μ, *l*,* r*). While analyses of rates are becoming more common in the literature (e.g., Brody, Lozier, & Dunne, 2013; Brody & Lozier, 2014, 2015; George, Lonsdale, Merlo, & Gobler, 2015; Gutiérrez‐Rodríguez et al., 2011; Lawrence & Menden‐Deuer, 2012; Lindemann & St John, 2014; Llort et al., 2015; Thomalla, Racault, Swart, & Monteiro, 2015; Venables et al., 2013; Westberry et al., 2016) and have provided some of the most interesting (and challenging!) recent findings, the practice of only reporting biomass data remains far too common and makes it near‐impossible to objectively identify key features in the annual cycle. To illustrate the problem, Figure 6a shows the modeled time series of mixed layer *C*_phyto_ over a period of water column stratification. Clearly, a bloom has been created by the end of the time series, but when was this bloom initiated? Some recent publications have required readers to identify initiation through simple visual inspection of *C*_phyto_ or chlorophyll data, which for the data in Figure 6a might be placed around day 80 (top black arrow). Other publications use a more quantitative criterion, such as *C*_phyto_ or chlorophyll exceeding the annual average or median value by 5%. For the data in Figure 6a, these definitions place initiation at day 69 (top blue arrow) and day 51 (top red arrow), respectively. In truth, our modeled bloom was created with a constant rate of accumulation (Figure 6b) and thus there was no specific initiation date. Similarly, we can apply these two criteria to a real annual cycle in *C*_phyto_ from the subarctic Atlantic, giving initiation dates in April and May, respectively (Figure 6c, red and blue arrows). However, actual values of *r* for this time series reveal that periods of blooming occurred from February through June (Figure 6d, yellow shading) and that no significant event is captured by either of the aforementioned criterion (Figure 6d, red and blue symbols).

So, here are the main points of this section. When reading the bloom literature, be aware of the commonalities and defining differences between hypotheses. For example, they all predict an increase in *C*_phyto_ with increasing mixed layer light (assuming ample nutrients), but some require thresholds while others do not. Also, consider the time frame of a given study with respect to the range of conditions encountered, as it is very difficult to conclusively demonstrate thresholds with temporally limited data. Finally, be cautious of conclusions drawn from time series in biomass when accumulation rates are not also provided. At the very least, biomass data should be log‐transformed to give a first‐order impression of rates of change, but studies directly reporting values of *r* are far easier to evaluate. Without such information, key events in the annual cycle (e.g., when blooming [i.e., positive *r*] occurs) can be grossly misrepresented and, consequently, incorrect conclusions will be drawn on the governing processes. On a similar note, it is important to evaluate how a given study handles the dilution effect of mixed layer deepening, as this simple physical process influences conclusions regarding the balance between μ and *l*.

## 14 THOUGHTS FOR THE FUTURE

To close this tutorial, we offer a few suggestions regarding future studies of phytoplankton blooms and annual biomass cycles. Obviously, one important recommendation is that the reporting of specific rates (μ, *l*, and *r*) becomes standard practice. Work is also needed to reconcile methodological differences in the assessment of these rates (e.g., ^14^C uptake, dilution experiments, etc.) (e.g., Landry et al., 2011; Marañon, 2005) and to ensure that measured values accurately capture *in situ* rates. Regarding metrics of standing stocks, the most commonly reported property has been bulk chlorophyll concentrations. Refocusing future work on *C*_phyto_ would be preferable, as chlorophyll can be strongly impacted by physiology (e.g., nutrient stress and light limitation responses) (e.g., Behrenfeld et al., 2016; Falkowski & LaRoche, 1991; Geider, 1987; Geider et al., 1998; Laws & Bannister, 1980) and organic carbon is the relevant “currency” with respect to trophic exchange. Westberry et al. (2016) demonstrated the significance of this issue by showing subarctic North Pacific blooms as having comparable climax values as the subarctic North Atlantic in terms of *C*_phyto_ but vastly different values in terms of chlorophyll. Another issue is whether standings stocks should be evaluated in terms of concentrations (e.g., mg C m^−3^) or abundances (e.g., cells/m^3^). For example, one tenet of the *Disturbance Recovery Hypothesis* is that equilibrium values of *C*_phyto_ covary with μ, which is inconsistent with *Prochlorococcus* populations in the central ocean gyres that have low biomass but relatively high μ (order 0.63 day^−1^ = 1 division per day) (Vaulot, Marie, Olson, & Chisholm, 1995). However, this inconsistency may be reconciled by replacing *C*_phyto_ in the aforementioned tenet with number abundance (the more relevant property with respect to predator–prey encounter rates and viral infection), which for *Prochlorococcus* is of order 10^5^ cells/ml in the gyres (e.g., Durand, Olson, & Chisholm, 2001; Winn et al., 1995). In addition to the above, we harken back to Gran's, 1912 statement, “One species succeeds another as month follows month,” and note that considerable work is still needed in resolving the role of species succession on phytoplankton annual cycles (Barber & Hiscock, 2006; Irigoien, Flynn, & Harris, 2005; Romagnan et al., 2015). Here, in particular, we may anticipate new understanding to parallel recent technological advances in genomics, optics, and imaging systems. Finally, work on the *Critical Turbulance Hypothesis* (Huisman et al., 1999, 2002; Taylor & Ferrari, 2011a, 2011b) and mesoscale dynamics (Mahadevan, D'Asaro, Lee, & Perry, 2012) has reminded us that the details of upper ocean physics are critically important in shaping *C*_phyto_ distributions and temporal developments.

Riley's work in the 1940s first demonstrated the power of numerical modeling in biological oceanography and this tool will undoubtedly continue to inform thought on the interplay between factors regulating phytoplankton division and loss rates. We draw one example here from our work on the *Disturbance Recovery Hypothesis*. Using monthly satellite data, we find that the coupling between μ and *l* occurs on a time scale of days (much as Riley 1946 suggested) and that values of *r* are one to two orders of magnitude smaller than μ (Behrenfeld & Boss, 2014; Behrenfeld et al., 2016, 2017). These relationships change significantly when monthly data are replaced by 8‐day satellite data. Specifically, the apparent coupling time between μ and *l* is longer and *r* exhibits higher frequency variability covering a greater range in values (e.g., Behrenfeld, 2010, 2014). Interestingly, variability in *r* at both the 8‐day and monthly time scales is well described by the rate of change in μ (i.e., dμ/d*t*) (Behrenfeld, 2014; Behrenfeld et al., 2016, 2017). Field data can provide even finer temporal resolution snapshots of the division–loss balance, at times indicating values of *r* approaching those of μ. Clearly, our view of ocean ecosystem functioning depends on the time resolution of our data and a challenge is to understand how processes at shorter time‐scales give rise to properties observed at longer time‐scales. Modeling can help here.

**Figure 7:**
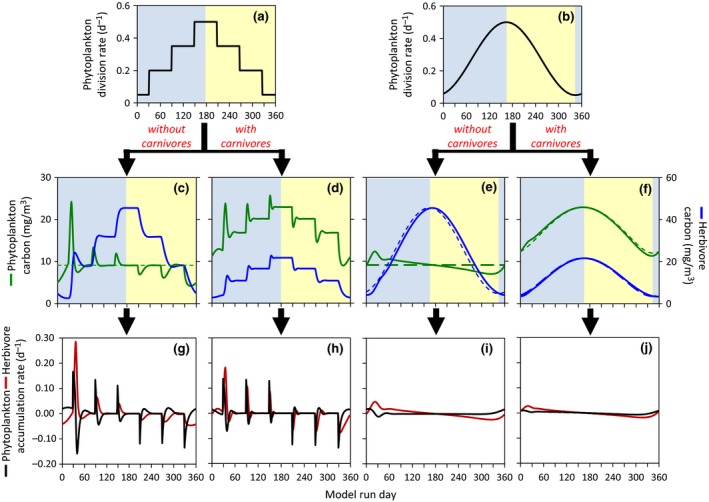
Annual phytoplankton and herbivore annual cycles from numerical model described by Behrenfeld and Boss (2014). For the four versions of the model, phytoplankton division rates were prescribed either as (a) step function in time or (b) a smooth sinusoidal function of time. (c–f) Time series of (green line) phytoplankton carbon concentration and (blue line) herbivore carbon concentration. Dashed lines in panels c, e, and f indicate equilibrium solutions. (g–j) Time series of carbon concentration accumulation rates for (black line) phytoplankton biomass and (blue line) herbivore biomass. (c, e, g, i) Model results where herbivore mortality is described as a linear function of concentration. (d, f, h, j) Model results where herbivore mortality includes both a linear function of concentration and a squared power of herbivore concentration (representing carnivory and other nonlinear loss processes). See also Appendix S2

As an illustration, we will employ a simple numerical model (Behrenfeld & Boss, 2014) that is similar to Riley (1946) and Evans and Parslow (1985) and executed in four versions (Figure 7). Comparable results are achieved using an even simpler model that can be analytically evaluated (Appendix S2). For the first two versions of our model, μ is prescribed in a stepwise manner, starting at a value of 0.05 day^−1^ and increasing every 60 days in increments of 0.15 day^−1^ up to a maximum of 0.50 day^−1^ (Figure 7a). After 60 days at this maximum rate, μ is then decreased every 60 days in 0.15 day^−1^ increments until the minimum of 0.05 day^−1^ is once again reached (Figure 7a). In the other two versions, μ is described as a smooth sinusoidal function with a period of 360 days (Figure 7b). A linear term for herbivore mortality is included in all four versions of the model, but in two versions a nonlinear term is also added to reflect loss processes dependent on encounter rates. This density‐dependent term may be thought of zooplankton “carnivory” (e.g., Moore, Doney, Kleypas, Glover, & Fung, 2002) although it could encompass additional processes such as viral infection.

The first model outcome of interest is that incremental increases in μ yield steady‐state, or “equilibrium,” *C*_phyto_, and herbivore biomass only after an initial pulse in *C*_phyto_ and subsequent overgrazing (Figure 7c,d). The opposite sequence occurs for a decrease in μ, which first results in overgrazing and then an increase in *C*_phyto_. These transient oscillations are a consequence of temporal lags between changes in *C*_phyto_, density‐dependent grazing, and herbivore mortality (Figure 7g,h). The magnitude of these oscillations and the time required to reach equilibrium is greater at lower μ (Figure 7c,d,g,h). Thus, the immediate response of *C*_phyto_ is dependent on the relative change in μ, rather than the absolute change. In nature, we might imagine these transient responses as phytoplankton and herbivore population changes following a storm or other short‐term disturbance in mixed layer growth conditions, such as transient surface stratification, changes in near‐surface turbulence, or eddy‐driven restratification.

Another outcome of the stepwise model is that equilibrium *C*_phyto_ values are invariant over an order of magnitude range in μ when only a linear herbivore mortality term is included (Figure 7c, dashed green line). Perhaps counter intuitively, these changes in μ are entirely expressed in herbivore biomass (Figure 7a). In contrast, an annual cycle in *C*_phyto_ emerges when density‐dependent carnivory is added to the model (Figure 7d). This annual cycle in *C*_phyto_ results because the overgrazing period is significantly dampened when carnivores are present (Figure 7h) compared to the case without carnivores (Figure 7g). Thus, density‐dependent feeding by carnivores causes a trophic cascade (Hessen & Kaartvedt, 2014; Pace, Cole, Carpenter, & Kitchell, 1999; Verity & Smetacek, 1996; Verity, Smetacek, & Smayda, 2002) where steady‐state *C*_phyto_ covaries linearly with μ. However, the resultant range in *C*_phyto_ (factor of ~2) is much smaller than the associated range in μ (factor of 10). This dampened annual cycle in *C*_phyto_ compared to μ is consistent with natural populations (e.g., Behrenfeld, 2014; Behrenfeld et al., 2016, 2017).

Implementing a smooth annual cycle in μ (Figure 7b) yields *C*_phyto_ and herbivore cycles (Figure 7e,f) that are to first order consistent with the stepwise model (Figure 7c,d), but with the important difference that equilibrium solutions are never quite achieved. This difference is what Evans and Parslow (1985) referred to as the “quasi‐equilibrium” solution. Continuous changes in μ cause *C*_phyto_ and herbivore biomass to be slightly above and below equilibrium values (Figure 7e,f, dashed lines), respectively, when μ is increasing and vice versa when μ is decreasing. These very subtle differences between the quasi‐equilibrium and equilibrium solutions imply a very strong attraction to steady‐state in highly coupled predator–prey ecosystems.

What this simple modeling exercise reveals is that the rate of change in environmental factors regulating μ plays a strong governing role in the temporal dynamics of *r*. When μ changes slowly in time, the annual cycle in *C*_phyto_ is driven by *r* values one to two orders of magnitude smaller than μ (Figure 7b,i,j). Similar results are found in monthly satellite data because temporal averaging dampens short‐term oscillations in environmental conditions (e.g., Figure 6d). Our stepwise simulations provide some insight on transient ecosystems responses to higher‐frequency environmental variability, briefly yielding *r* values of similar order as μ (Figure 7a,g,h) that might be captured in field or 8‐day resolution satellite observations. Thus, modeling can provide useful insights for both reconciling apparent divergences between studies and guiding the design of future studies. For example, one might consider targeting a location and time where storm frequency allows multiple predator–prey coupling and de‐coupling events to be captured within a reasonable time‐frame for ship observations. One might alternatively consider how disturbance events might be artificially created in natural plankton assemblages.

Our modeling results also highlight a need for understanding how variations in *C*_phyto_ are linked to trophic cascade processes. Progress along these lines has certainly been realized in experimental lake studies (Sterner & Elser, 2002), but how might such approaches be extend to open ocean bloom‐forming regions and over annual times scales? Finally, modeling studies (Auger et al., 2014; Behrenfeld & Boss, 2014; Behrenfeld et al., 2013; Lindemann & St John, 2014; Llort et al., 2015), in addition to satellite (Behrenfeld, 2010, 2014; Behrenfeld et al., 2016, 2017; Brody et al., 2013; Brody & Lozier, 2014; Navarro et al., 2012) and field data (Boss & Behrenfeld, 2010; Daniels et al., 2015; D'Ortenzio et al., 2014; George et al., 2015; Venables et al., 2013), have clearly shown the importance of evaluating blooms in the context of complete phytoplankton annual cycles. These findings accordingly suggest a shift in future field studies away from the bloom climax period and more during times of the year historically not associated with blooming. For example, sustained measurements throughout winter in the North Atlantic and encompassing periods of convective mixed layer deepening and transitions to positive net heat flux could be instrumental to our understanding of factors influencing the phytoplankton division–loss balance.

In closing, we hope this tutorial has given some interesting historical accounts, clarified basic hypotheses, and provided useful tools and a framework for thinking about blooms and reading the bloom literature. In your work on plankton ecology, it is always worth remembering that across the global ocean, phytoplankton standing stocks are everywhere and always a reflection of the balance between division rates and the summation of all losses (grazing, viral lysis, sinking, cell death, physical mixing, etc.). These loss processes, in turn, are tightly linked to variations in phytoplankton stocks and have dependencies on higher trophic levels (e.g., carnivory on herbivores). Phytoplankton stocks and their rates of change are also undeniably dependent on “bottom‐up” factors controlling division (e.g., nutrients, temperature, light). These growth limiting factors are controlled by aspects of the physical environment that are directly impacted by climate change. The complex relationships governing phytoplankton biomass are perhaps most dramatically expressed through the annual recreation of seasonal blooms, making an understanding of blooms a fundamental challenge in global change biology (Martinez, Raitsos, & Antoine, 2016). As we address this challenge, we can hope that traditional and current hypothesis on blooms will be shed or fruitfully transformed. Science is always a work in progress and our publications mere progress reports on an endless journey toward understanding. Whether finishing our graduate degree or finishing our career, we all remain students.

## CONFLICT OF INTEREST

The authors have no conflict of interest to declare.

## Note

1 Here and below, we have retained units from original texts. An approximate conversion to standard units is as follows: 277.32 g cal cm^−2^ min^−1^ = mole quanta m^−2^ day^−1^.
