# Supplementary material for: Student's tutorial on bloom hypotheses in the context of phytoplankton annual cycles
Source: Glob Chang Biol. 2017 Sep 22;24(1):55–77. doi: 10.1111/gcb.13858 (PMC5763361; doi:10.1111/gcb.13858)
Supplement: Supplementary file 1 [file GCB-24-55-s001.pdf]

Supporting Information

for

Behrenfeld & Boss

Student's tutorial on bloom hypotheses in the context of phytoplankton annual cycles

Appendix A

In this appendix, we compute how a phytoplankton population experiencing an annual cycle in growth rate will develop in time for three different assumed loss rate formulations, as described in the tutorial and in Fig. 6. In *all* three cases, we assume a sinusoidal time-varying growth rate:

$$\mu = \mu_0 + \mathrm{A}\sin\left(\frac{2\pi t}{T} - \varphi\right),$$

where T is one year, φ is a phase lag such that the maximum growth rate occurs at the right month of the year (e.g. June). We vary the formulation of the loss rate, l, and solve analytically the phytoplankton conservation equation (neglecting ML dynamics and sinking):

$$\frac{dp}{dt} = (\mu - l)p.$$

Case 1 – constant annual loss rate (corresponds to Fig. 6b):

In this case, the phytoplankton biomass equation can be written as:

$$\frac{dp}{dt} = (\mu - l)p = \left\{\mu_0 - l + \mathrm{A}\sin\left(\frac{2\pi t}{T} - \varphi\right)\right\}p.$$

The solution of which is achieved by simple integration in time and is:

$$p(t) = p(0)exp\left\{(\mu_0 - l)\mathrm{t} - \frac{\mathrm{AT}\cos}{2\pi}\left(\frac{2\pi t}{T} - \varphi\right)\right\}.$$

The part of the exponential that is linear in time will result in either unrealistic large populations ($\mu_0 > l$) or a decimation of the population ($\mu_0 < l$) unless $\mu_0 = l$ exactly, which is an unrealistic constraint.

Case 2 – loss rate varying linearly with growth rate (corresponds to Fig. 6c):

The loss rate is assumed to be a linear function of growth rate:

$$l = B\mu + C.$$

For this scenario:

$$\frac{dp}{dt} = (\mu - l)P = \left\{\mu_0(1 - B) - \mathrm{C} + \mathrm{A}(1 - B)\sin\left(\frac{2\pi t}{T} - \varphi\right)\right\}$$

whose solution is:

$$p(t) = p(0)exp\left\{(\mu_0(1 - B) - C)\mathrm{t} - \frac{\mathrm{A}(1-B)\mathrm{T}\cos}{2\pi}\left(\frac{2\pi t}{T} - \varphi\right)\right\}.$$

For this solution to not grow or decay exponentially in time (as above) the condition $(\mu_0(1 - B) - C) = 0$ has to be satisfied. In that specific case, $\mu, l,$ and $r = \mu - l,$ are all in phase, but 3 months out of phase with $p(t)$, inconsistent with observations.

Case 3: **loss rate slightly out of phase with growth rate (corresponds to Fig. 6d)**:

The loss rate follows a similar function as growth rate with a constant time lag relative to it:

$$l = \mu_0 + \mathrm{A}\sin\left(\frac{2\pi t}{T} - \varphi - \Delta\varphi\right).$$

In this case:

$$\frac{dp}{dt} = (\mu - l)P = \left\{\mathrm{A}\sin\left(\frac{2\pi t}{T} - \varphi\right) - \mathrm{A}\sin\left(\frac{2\pi t}{T} - \varphi - \Delta\varphi\right)\right\}p = 2A cos\left(\frac{2\pi t}{T} - \varphi - \frac{\Delta\varphi}{2}\right) sin\left(\frac{\Delta\varphi}{2}\right)p,$$

whose solution is:

$$p(t) = p(0)exp\left\{\frac{sin\left(\frac{\Delta\varphi}{2}\right)\mathrm{AT}}{\pi}\left[sin\left(\frac{2\pi t}{T} - \varphi - \frac{\Delta\varphi}{2}\right) + sin\left(\varphi + \frac{\Delta\varphi}{2}\right)\right]\right\} =$$
$$p(0)exp\left\{sin\left(\varphi + \frac{\Delta\varphi}{2}\right)\right\} exp\left\{\frac{sin\left(\frac{\Delta\varphi}{2}\right)\mathrm{AT}}{\pi}\left[sin\left(\frac{2\pi t}{T} - \varphi - \frac{\Delta\varphi}{2}\right)\right]\right\}.$$

This solution is a periodic function displaying an annual cycle *in phase* with growth and loss-rates. The solution depends on $\Delta\varphi$. For the maximum in biomass, growth, and loss to occur near the same time and for the phytoplankton winter to summer biomass change to be constrained within realistic values, $\Delta\varphi$ has to be small (e.g. a few days).

Conclusion: In this appendix, we explored the consequences of different formulations of loss rates as a function of the growth rate. We find that each has significantly different behavior. In the main text, we argue that case 3 results in the most realistic behavior despite its simplistic formulation. All of these solutions lead to testable hypotheses that can be supported or refuted with appropriate observations.

Appendix B

In this appendix, we analyze a very simple prey-predator model that has the basic features of the models we used in Behrenfeld and Boss (2014) and in the tutorial. As Evans and Parlow (1983) demonstrated, such models are very useful to diagnose the time varying steady-state points named quasi-steady state, which are the 'attractors' for the solution.

We assume a very simple ecosystem model with two compartments, phytoplankton (P) and herbivores (H), and an upper ocean of constant mixing depth (MLD). The solutions presented below are for concentrations of phytoplankton and herbivores and are independent from the mixed-layer depth (except where the latter affects α).

We denote by α the phytoplankton net growth. Grazing is represented by c_1PH, which is ingested into herbivores with efficiency c_2. Herbivore mortality is parametrized by a linear term ('natural death', c_3H) and a non-linear term ('carnivory', c_4H^2). The resulting system of equation is:

$$\frac{dP}{dt} = \alpha P - c_1 PH$$

(1)

$$\frac{dH}{dt} = c_2 c_1 PH - c_3 H - c_4 H^2$$

When $c_4 = 0$, this system is essentially the Lotka-Voltera equations (e.g. Murray, 2002). In that specific case, the steady-state solutions are the unstable and trivial solution, $(P_0, H_0) = (0,0)$, and a limit cycle oscillation around $(P_0, H_0) = \left(\frac{c_3}{c_2 c_1}, \frac{\alpha}{c_1}\right)$, whose period is $T = \frac{2\pi}{\sqrt{\alpha c_3}}$ and whose amplitude depends on the initial conditions.

For literature values of these parameters (e.g. Laws, 2013: $\alpha \sim 0.7 d^{-1}$, Evans and Parslow, 1983, $c_3 \sim 0.07\ d^{-1}$) the period is O (28 days). A limit-cycle behavior is not consistent with observations, as we do not observe a continuously alternating phytoplankton-herbivore dominance change. Furthermore, the phytoplankton concentration around which the oscillation occurs is independent of growth-rate, and observations suggest that phytoplankton are more abundant the larger their growth-rate.

Assuming a non-zero 'carnivory' term ($c_4 \neq 0$) makes the steady-state solution more realistic. The none zero steady-state of this system is stable (an attractor) and both phytoplankton and herbivore concentrations in steady-state depend on phytoplankton growth rate,

$$(P_0, H_0) = \left(\frac{c_3}{c_2 c_1} + \frac{\alpha c_4}{c_2 c_1^2}, \frac{\alpha}{c_1}\right).$$

Note, however, that while herbivore concentration is directly proportional to growth rate (will double when growth rate doubles), the concentration of phytoplankton is linearly related to it (will not double when growth rate doubles).

The convergence rate to the steady state (the e-folding time) is $\frac{c_1}{c_4 \alpha} d$. This means that the steady-state solution will be reached faster with increasing growth rate and herbivore loss-to-carnivory rate, but will decrease with grazing rate. For literature values of these parameters (e.g. Laws, 2013: $\alpha \sim 0.7 d^{-1}$, Evans and Parslow, 1983: $c_1 \sim 1\ (mmol\ N\ m^{-3})^{-1}\ \ d^{-1}$, Moore et al., 2002: $c_4 \sim 0.06\ (mmol\ N\ m^{-3})^{-1}\ \ d^{-1}$), the convergence time is 24 days.

Note that the specific location of the steady-states in the (P, H) space varies as the parameters change in time (in particular the net growth rate α). As long as the changes in these parameters are long compared to the convergence rate, there is the possibility that the ecosystem is near steady-state. Also note that one can use this analysis to constrain parameters with observations; α, P, and convergence rates following a perturbation (storm) can be constrained with space and in-situ observations.

References

Evans GT, Parslow JS (1985) A model of annual plankton cycles. *Biol. Ocean*. **3**, 327-347.

Moore JK, Doney SC, Kleypas JA, Glover DM, Fung IY (2002) An intermediate complexity marine ecosystem model for the global domain. *Deep-Sea Res. II* **49**, 403-462.

Murray JD (2002) *Mathematical Biology*, Springer.

Laws E (2013) Evaluation of in situ phytoplankton growth rates: A synthesis of data from varied approaches. *Annu. Rev. Marine. Sci*. 5, 247-268
